# Controlling the regioselectivity and stereospecificity of FAD-dependent polyamine oxidases with the use of amine-attached guide molecules as conformational modulators

**DOI:** 10.1042/BSR20180527

**Published:** 2018-08-29

**Authors:** Tuomo A. Keinänen, Nikolay Grigorenko, Alex R. Khomutov, Qingqiu Huang, Anne Uimari, Leena Alhonen, Mervi T. Hyvönen, Jouko Vepsäläinen

**Affiliations:** 1 School of Pharmacy, Biocenter Kuopio, University of Eastern Finland, Kuopio Campus, P.O. Box 1627, Kuopio FI-70211, Finland; 2 MacCHESS at the Cornell High Energy Synchrotron Source, Cornell University Ithaca, NY 14853-8001, U.S.A.; 3 BASF Schweiz AG, Dispersions and Pigments Division, Klybeckstrasse 141, P.O. Box CH 4002, Basel, Switzerland; 4 Engelhardt Institute of Molecular Biology, Russian Academy of Sciences, Vavilov St 32, Moscow 119991, Russia; 5 Natural Resources Institute Finland, Natural Resources Division, Neulaniementie 5, Kuopio FI-70210, Finland

**Keywords:** amine oxidoreductase, biocatalysis, chirality, drug discovery and design, flavins

## Abstract

Enzymes generally display strict stereospecificity and regioselectivity for their substrates. Here by using FAD-dependent human acetylpolyamine oxidase (APAO), human spermine (Spm) oxidase (SMOX) and yeast polyamine oxidase (Fms1), we demonstrate that these fundamental properties of the enzymes may be regulated using simple guide molecules, being either covalently attached to polyamines or used as a supplement to the substrate mixtures. APAO, which naturally metabolizes achiral *N*^1^-acetylated polyamines, displays aldehyde-controllable stereospecificity with chiral 1-methylated polyamines, like *(R)-* and *(S)-*1-methylspermidine (1,8-diamino-5-azanonane) (1-MeSpd). Among the novel *N*^1^-acyl derivatives of MeSpd, isonicotinic acid (P4) or benzoic acid (Bz) with *(R)*-MeSpd had *K*_m_ of 3.6 ± 0.6/1.2 ± 0.7 µM and *k*_cat_ of 5.2 ± 0.6/4.6 ± 0.7 s^−1^ respectively, while *N^1^*-AcSpd had *K*_m_ 8.2 ± 0.4 µM and *k*_cat_ 2.7 ± 0.0 s^−1^. On the contrary, corresponding *(S)*-MeSpd amides were practically inactive (*k*_cat_ < 0.03 s^−1^) but they retained micromole level *K*_m_ for APAO. SMOX did not metabolize any of the tested compounds (*k*_cat_ < 0.05 s^−1^) that acted as non-competitive inhibitors having *K*_i_ ≥ 155 µM for SMOX. In addition, we tested *(R*,*R)*-1,12-bis-methylspermine (2,13-diamino-5,10-diazatetradecane) *(R*,*R)-*(Me_2_Spm) and *(S,S)*-Me_2_Spm as substrates for Fms1. Fms1 preferred *(S,S)*- to *(R,R)*-diastereoisomer, but with notably lower *k*_cat_ in comparison with spermine. Interestingly, Fms1 was prone to aldehyde supplementation in its regioselectivity, i.e. the cleavage site of spermidine. Thus, aldehyde supplementation to generate aldimines or *N*-terminal substituents in polyamines, i.e. attachment of guide molecule, generates novel ligands with altered charge distribution changing the binding and catalytic properties with polyamine oxidases. This provides means for exploiting hidden capabilities of polyamine oxidases for controlling their regioselectivity and stereospecificity.

## Introduction

**Figure 1:**
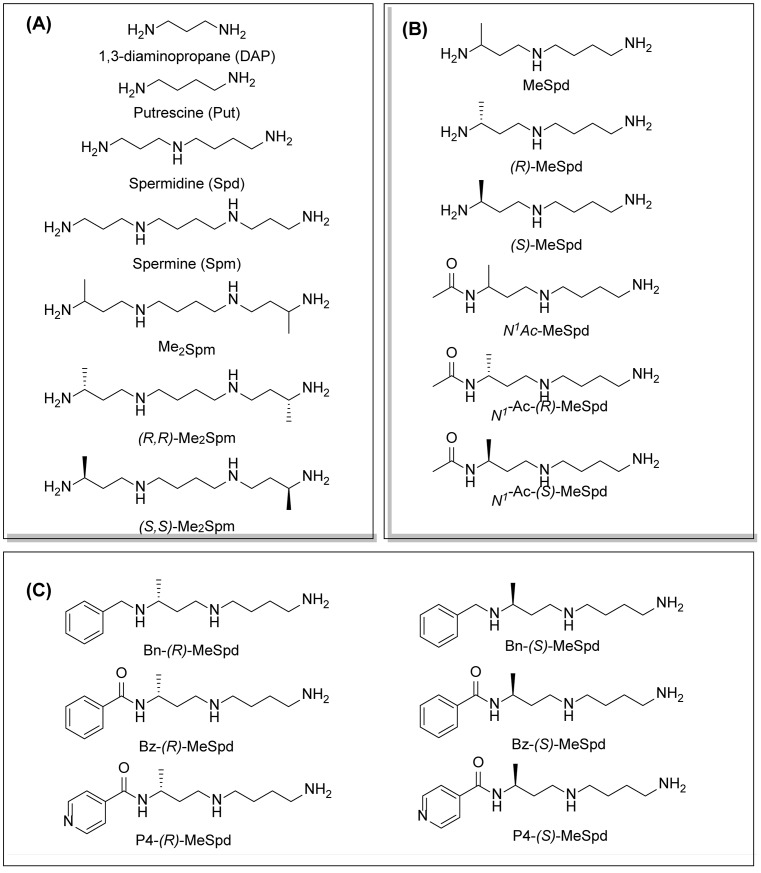
Chemical structures of the reference and tested compounds Structures of (**A**) 1,3-Diaminopropane (DAP), natural polyamines and dimethylated analogues of Spm. (**B**) 1-Methylated spermidine analogues and their *N^1^*-acetylated derivatives. (**C**) Guide molecule-derivatives of *(R)*-MeSpd and *(S)*-MeSpd. Abbreviation: MeSpd, 1-methylspermidine (1,8-diamino-5-azanonane).

The polyamines spermidine (Spd) and spermine (Spm) and their diamine precursor putrescine (Put) are essential cellular constituents in eukaryotic organisms [1] (Figure 1A). Their intracellular levels are strictly regulated by *de novo* synthesis, active transport, excretion and catabolism by a complex cellular regulatory network [2,3]. Interconversion of Spm into Spd is enzymatically regulated by FAD-dependent spermine oxidase (SMOX; EC 1.5.3.16) or by consequent actions of Spd/Spm-*N^1^*-acetyltransferase (SSAT; EC 2.3.1.57) and acetylpolyamine oxidase (APAO; EC 1.5.3.13) [4,5]. Recent studies clearly show that polyamine metabolism is disturbed in a variety of diseases or medical disorders, such as cancer, brain insult and diabetes [6,7]. Furthermore, polyamine metabolism differs between parasites, microbes and the host, which could be used for developing novel therapies [8].

Oxidative catabolism of polyamines generates acrolein and reactive oxygen species (ROS) like hydrogen peroxide, which in excess are harmful to cells. Dysregulation of SMOX and activated Spm catabolism are associated with inflammation-mediated development of cancer [9]. There is direct evidence that the induction of SMOX during neoplastic transformation leads to the development of colon and gastric cancer. Furthermore, in cancer cells APAO has been shown to detoxify *N*-alkylated polyamine analogues [10], while induction of SMOX is responsible for the toxic effects of *N*-alkylated polyamine analogues [11]. Thus, APAO and SMOX sometimes play opposite roles in determining drug sensitivity of cancer cells. So far, determinations of crystal structure of native APAO and SMOX have been unsuccessful, although recently several crystal structures of slightly mutated murine APAO were reported [12]. The latter data in combination with the available yeast polyamine oxidase (Fms1) and maize PAO crystal structures, computer modelling and experiments with targeted point mutations into recombinant proteins have been used to study the possible structure-activity determinants of APAO and SMOX [13–16]. All the previous enzymes are available as recombinant proteins and their structure-activity properties *in vitro* have been relatively well characterized. Unfortunately, obtaining highly selective small-molecule inhibition of either APAO or SMOX has been unsuccessful, leaving gene silencing as the only viable option to investigate the physiological functions of these enzymes [17].

α-Methylation is an efficient chemical modification to protect amine-based drugs against degradation by cellular mono- and diamine oxidases and to modulate drug ADME properties [18,19]. Some of the α-methylated drug derivatives have proved to be efficient inhibitors of parent oxidases that catabolize biogenic amines [18]. Racemic α-methylated polyamines 1-methylspermidine (1,8-diamino-5-azanonane) (MeSpd), MeSpm and 1,12-bis-methylspermine (2,13-diamino-5,10-diazatetradecane) (Me_2_Spm) were synthesized by Lakanen et al. [20] (Figure 1A/B). They were shown to be metabolically stable, i.e. not acetylated by SSAT with the exception of MeSpm, and were able to substitute natural polyamines in supporting cell growth under natural polyamine deprivation [20,21]. MeSpd and Me_2_Spm are not so readily metabolized *in vivo* as Spd and Spm, and *in vitro* they are not catabolized to toxic compounds by serum amine oxidases [20,22]. Thus, they seem to be ideal candidates for *in vivo* use [23,24]. Although natural polyamines are achiral, we have discovered the hidden stereospecificity of APAO, SMOX and deoxyhypusine synthase (DHS; 2.5.1.46) [24–26]. APAO preferably oxidizes the *(R)*-enantiomer of *N*^1^-Ac*-*MeSpd [24]. *(S,S)*-Me_2_Spm is a substrate of SMOX while *(R,R)*-Me_2_Spm is not metabolized by the enzyme [25], and *(S)*-MeSpd is a source of aminobutyl fragment in DHS reaction [26]. Furthermore, we have recently shown that polyamine transport system and the key enzymes of polyamine metabolism, namely ornithine decarboxylase (ODC), *S*-adenosyl-*L*-methionine decarboxylase (AdoMetDC) and SSAT are divergently regulated by chiral *C*-methylated polyamine analogues [27,28]. Our earlier findings indicate that the stereospecificity of FAD-dependent human APAO can be altered with the aid of simple guide molecules [29]. Guide effects of aromatic aldehydes in APAO reaction using racemic MeSpd as a substrate were very clear and unexpected. Benzaldehyde stimulated the splitting of *(R)*-MeSpd, pyridoxal—splitting of *(S)*-MeSpd, while 4-pyridinealdehyde was not able to induce stereospecificity [29]. All above prompted us to synthetize a set of earlier unknown *N*^1^-benzylated (Bn) or *N*^1^-acylated, i.e. isonicotinic acid (P4) and benzoic acid (Bz) amide derivatives of *(R)*- and *(S)*-MeSpd to further explore characteristics of FAD-dependent amino oxidoreductases (Figure 1C).

Here we studied the substrate specificities of SMOX and APAO for *N^1^*-alkylated or *N*^1^-acylated derivatives of *(R)*- and *(S)*-MeSpd and the effects of supplemented aldehydes on Fms1, that readily catalyses the oxidation of *N^1^*-acetylated Spd and Spm. We also used *(R,R)*-Me_2_Spm and *(S,S)*-Me_2_Spm to gain insight into how 1,12-bis-methylation of Spm and configuration of chiral centres affects the substrate properties and binding to the active centre of Fms1 (Figure 1A). *N^1^*-Acetylated derivatives of 1-MeSpd were synthesized to complete the series of analogues, tested with the Fms1 and to compare the results with the known stereospecificity of APAO (Figure 1B). Obtained data demonstrate for the first time that stereospecificity and regiospecificity of FAD-dependent polyamine oxidases could be controlled with the conformationally restricted ligands exploiting existing conformational landscapes in enzyme without protein engineering.

## Experimental procedures

### Materials

All the commercially available chemicals were purchased from Sigma–Aldrich. *(R,R)*-Me_2_Spm, *(S,S)*-Me_2_Spm and racemic Me_2_Spm, *(R)*-MeSpd and *(S)*-MeSpd enantiomers and their covalently modified guide molecule derivatives were synthesized essentially as described in [24].

### Production of recombinant enzymes and enzyme tests

The production of human recombinant APAO, SMOX and yeast Fms1 has been described earlier [16,22]. Substrate and aldehyde supplement concentrations and experimental conditions are described in Figures and Tables captions. HPLC with post-column *o*-phthalaldehyde-derivatization was used to determine the concentrations of the reaction products Put and 1,3-diaminopropane (DAP) or butane-1,3-diamine respectively as described in [30]. Fms1 activity was determined essentially as described for human recombinant APAO, but reactions were carried out in 100 mM Glycine-NaOH buffer at pH 9.0 in a water bath at +25°C [29,30]. Reactions for kinetic value determinations were carried out at pH 9.0 in 100 mM Glycine-NaOH in triplicates by using 25, 50, 75, 100, 200, 400 and 600 µM substrate concentrations for Spm and for Me_2_Spm but 600 µM concentration was replaced with 1 mM concentration in Me_2_Spm series. Kinetic values were determined by using Michaelis–Menten equation and non-linear regression by using GraphPad Prism software 5.03 with enzyme kinetic template. Fms1 activity compared with pH was determined by using 1 mM Spm with 0.1 µg of Fms1 in 170 mM Bis/Tris buffer at pH 7.4, 8.0, 8.25, 8.5, 8.75, 9.0, 9.25 and 9.5 incubated 4 min at 25°C. *k*_cat_ values were determined using an *M*_r_ of 55382 for human recombinant APAO, *M*_r_ 62000 for SMOX and for Fms1 using *M*_r_ of 58833 [31].

*K*_i_ values for covalently modified MeSpd derivatives for SMOX were determined as triplicates using at least four inhibitor concentrations (25, 100, 200, 250, 500, 1000 or 2000 µM) in the presence of 25, 50 or 100 µM Spm. Reaction mixtures contained 40 units/ml horseradish peroxidase (Roche), 1 mM homovanillic acid in 100 mM Glycine-NaOH at pH 9.0 supplemented with 40 ng of SMOX. The reaction kinetics were monitored at 37°C using excitation at 315 nm and emission at 420 nm using Envision spectrofluorometer (PerkinElmer). Dilutions of fresh H_2_O_2_ were used as standard. GraphPad Prism 5.03 software using non-competitive non-linear Michaelis–Menten fitting was used to determine *K*_i_ values.

### Preparation of rat liver extract

A Wistar rat liver was frozen in liquid nitrogen. The liver was homogenized (1+3 w/v) with Teflon potter in buffer containing 25 mM Tris/HCl pH 7.4, 1 mM DTT and 0.1 mM EDTA. Resulting homogenate was centrifuged at 12000×***g*** for 30 min at +4°C. Supernatant was divided into two portions and treated as follows: (A) incubated for 5 min at +37°C in a water bath, (B) supplemented with 20 μM MDL 72527 and incubated for 5 min at +37°C in a water bath to inactivate APAO and SMOX. A 20-μl aliquot of supernatant A or B was added in 100 mM Glycine-NaOH pH 9.5, 5 mM DTT with or without 100 μM of studied drug in a total volume of 180 μl. After 10-min incubation at +37°C, 20 μl of 50% sulphosalicylic acid (SSA) containing 100 μM diaminoheptane (DAH) was added to the reaction mixture. The samples were assayed with HPLC as described in [30].

## Results and discussion

### *N*-acylated and *N*-alkylated derivatives of *(R)*- and *(S)*-MeSpd as substrates of human recombinant APAO

**Figure 2:**
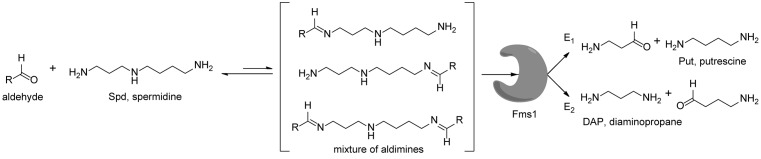
Simplified sketch showing chemical principle for using aldehyde supplementation to generate *in situ* aldimines mimicking the charges of *N*-acetylated Spd species In aqueous solution, equilibrium is strongly favouring free Spd and aldehyde species. However, by increasing aldehyde concentration it is possible to increase aldimine pool concentration, e.g. Table 4 and accelerate Fms1-mediated degradation of Spd pool.

*N^1^*-acetylated derivatives of Spd and Spm are natural substrates of APAO and it has been shown that in the presence of aromatic aldehydes APAO efficiently metabolizes non-acetylated Spm and Spd. We have shown that the stimulatory effect of aldehydes on the APAO-catalysed oxidation of the polyamines is based on the *in situ* formation of comparatively unstable Schiff base between the primary amino group of the polyamine and the aldehyde, i.e. an aldimine mimicking the charge distribution of *N*-acetylated polyamines (Figure 2) [29,32]. Here we synthesized a set of novel chemically stable analogues of *N^1^*-AcSpd mimicking *in situ* formed Schiff base derivatives of 1-MeSpd enantiomers (Figure 1C) and tested them as substrates of APAO. As shown in Table 1, the *(R)*-enantiomers of these derivatives served as excellent substrates for recombinant human APAO. P4-*(R)*-MeSpd and Bz-*(R)*-MeSpd displayed enhanced catalytic velocity over the natural substrate *N^1^*-AcSpd. Interestingly, the respective *(S)*-enantiomers, P4-*(S)*-MeSpd and Bz-*(S)*-MeSpd, retained low *K*_m_ for APAO but practically lost their substrate properties, which renders them efficient competitive inhibitors. Amide derivatives P4-*(R)*-MeSpd and Bz-*(R)*-MeSpd were catalytically superior to Bn-*(R)*-MeSpd. Both Bn-*(R)*-MeSpd and Bn-*(S)*-MeSpd retained good affinity for APAO and the (*R)*-enantiomer displayed only five-fold higher *k*_cat_ than the *(S)*-enantiomer.

**Table 1:** Kinetic values of guide molecule-containing derivatives of MeSpds’ with human recombinant APAO

Polyamine	*K*_m_ (μM)	*V*_max_ (μmol/min/mg)	*k*_cat_ (s^−1^)	*k*_cat_/*K*_m_ (M^−1^ s^−1^)
*N*^1^-AcSpd^1^	8.2 ± 0.4	2.97 ± 0.02	2.7 ± 0.0	(330 ± 16) × 10^3^
Bz-*(R)*-MeSpd^2^	1.2 ± 0.7	5.02 ± 0.74	4.6 ± 0.7	(3800 ± 230) × 10^3^
Bz-*(S)*-MeSpd^2^	0.2 ± 0.2	0.03 ± 0.00	0.03 ± 0.00	(150 ± 150) × 10^3^
P4-*(R)*-MeSpd^3^	3.6 ± 0.6	5.59 ± 0.60	5.2 ± 0.6	(1400 ± 300) × 10^3^
P4-*(S)*-MeSpd^3^	0.8 ± 0.2	0.02 ± 0.00	0.01 ± 0.00	(18 ± 3.1) × 10^3^
Bn-*(R)*-MeSpd^4^	2.0 ± 0.1	0.15 ± 0.00	0.14 ± 0.00	(71 ± 3.5) × 10^3^
Bn-*(S)*-MeSpd^4^	1.6 ± 0.6	0.03 ± 0.00	0.03 ± 0.00	(18 ± 7.1) × 10^3^

Reactions were carried out three times in duplicates in 100 mM Glycine-NaOH at pH 9.5 supplemented with 5 mM DTT. Kinetic values were determined using GraphPad Prism 4.03 software using Michaelis–Menten equation with non-linear fitting (Supplementary Material 2). *k*_cat_ values were determined using an *M*_r_ of 55.382 for human recombinant APAO.

1 10, 25, 50, 75, 100, 200 μM concentrations were used.

2 2.5, 5, 7.5, 10, 25 μM concentrations were used.

3 2.5, 5, 7.5, 10, 25, 100 μM concentrations were were used.

4 5.0, 7.5, 10, and 25 μM concentrations were used.

We and others have shown earlier that the resistance of racemic 1-MeSpd for APAO-mediated degradation is due to the fact that SSAT is incapable of *N^1^*-acetylating it [20,24]. This was confirmed by using chemically synthesized *N^1^*-Ac-*(R)*-MeSpd (*K*_m_ = 95 µM, *k*_cat_ = 9 s^−1^) and *N*-Ac-*(S)*-MeSpd (*K*_m_ = 170 µM, *k*_cat_ = 1.2 s^−1^)—the former *(R)*-enantiomer is preferably metabolized by APAO [24]. Comparisons of their specificity constants, i.e. *k*_cat_/*K*_m_ of *N*-Ac-*(R)*-MeSpd (94737 M^−1^ s^−1^) and *N*-Ac-*(S)*-MeSpd (7059 M^−1^ s^−1^) for APAO with P4-*(R)*-MeSpd and P4-*(S)*-MeSpd having bulkier substituents show that the specificity constant ratio of *N*^1^-Ac*-(R)*-MeSpd/*N*^1^-Ac-*(S)*-MeSpd is only 13 in comparison with 116 with P4-*(R)*-MeSpd/P4-*(S)*-MeSpd derivatives. This explains why Schiff base formed by bulky aldehydes, like pyridoxal and benzaldehyde, allows almost complete catalytic activation of either (*S)*- or *(R)*-MeSpd respectively [29]. Surprisingly, the specificity constant ratio with Bn-*(R)*-MeSpd and Bn-*(S)*-MeSpd was only four in comparison with earlier determined eight for benzaldehyde Schiff base derivatives of *(R)*-MeSpd and *(S)*-MeSpd for APAO (Table 1) [29]. Importantly, among the amide derivatives, i.e. P4-MeSpd and Bz-MeSpd, we found only (*R)*-enantiomer-activating guide molecules showing specificity constant ratios of 116 and 25 respectively (Tables 1 and 2). Our present data show that in the case of MeSpd it is possible to regulate the substrate properties of APAO by changing the stereoconfiguration of chiral centre in combination with the structure of an attached *N*-acyl/*N*-alkyl substituent. These features could be exploited in drug design by generating *N*-alkylated polyamine analogues that are resistant against APAO/SMOX-mediated degradation. Furthermore, specific inhibitors or substrates for enzymatic assays for APAO could be prepared accordingly.

**Table 2:** Degradation of *N*^1^-AcSpd and *(R)*- and *(S)*-enantiomers of *N*^1^-substituted MeSpd in rat liver supernatant

Sample	Formation of polyamine *(pmol/mg protein)*		
	Put	Spd	Spm
0 min	ND	3024 ± 89	2875 ± 94
10 min	ND	2964 ± 36	2793 ± 34
*N*^1^-AcSpd 0 min	ND	3594 ± 18	3172 ± 30
*N*^1^-AcSpd 10 min	4175 ± 278	3480 ± 12	3049 ± 23
*N*^1^-AcSpd + MDL72527 10 min	ND	3436 ± 42	2984 ± 27
Bz-*(R)*-MeSpd 10 min*	5585 ± 288	2988 ± 2	3024 ± 18
P4-*(R)*-MeSpd 10 min*	8882 ± 66	2737 ± 78	3004 ± 74
Bn-*(R)*-MeSpd 10 min*	637 ± 13	3031 ± 72	2880 ± 234

Compounds were tested at 100 µM, which equalled 23000 pmol of the compound/mg of protein in the beginning of the reaction. Data are average of three individual reaction mixtures ± S.D. No detectable degradation of any of the tested compounds was found in the presence of MDL72527 (preincubation for 5 min before addition of the compound). Protein content of obtained liver homogenate was 39.2 µg/µl. Abbreviation: ND, not detectable.

* *(S)*-enantiomer derivatives were not degraded by rat liver homogenate under the experimental conditions used.

### *N*-alkylated and amide derivatives of *(R)-* and *(S)*-MeSpd as substrates of human recombinant SMOX

SMOX was cloned in 2001 [5,33] and was soon shown to be a distinct enzyme from the earlier characterized APAO [34]. SMOX has several splice variants among which at least two are catalytically active, one being cytosolic and the other showing cytosolic/nuclear localization [35,36]. Interestingly, many *N*-alkylated polyamine analogues induce SMOX, and induction of SMOX has been attributed to analogue-mediated growth inhibition and cytotoxicity [11]. Moreover, recent data clearly show that SMOX induction is associated with the development of gastric, prostate and colon cancers [37–39]. Thus, developing specific inhibitors of SMOX is of crucial importance [40]. In addition, the use of specific substrates for SMOX and APAO would enable distinguishing between APAO and SMOX enzyme activities *in vivo*. All the tested amide analogues had *K*_m_ over 100 µM and *k*_cat_ below 0.05 s^-1^. The *K*_i_ values for SMOX were 589 ± 58 µM for Bz-*(R)*-MeSpd, 846 ± 82 µM for Bz-*(S)*-MeSpd, 1277 ± 111 µM for P4-*(R)*-MeSpd and 1016 ± 79 µM for P4-*(S)*-MeSpd. Bn-*(S)*-MeSpd had *K*_i_ of 155 ± 13 µM and Bn-*(R)*-MeSpd *K*_i_ of 441 ± 32 µM, thus not being substrates of SMOX. The data on the interaction of acyl derivatives of *(R)*- and *(S)*-MeSpd, i.e. (Bz) and (P4) derivatives as well as alkyl (Bn) derivatives of MeSpd with APAO in comparison with SMOX clearly demonstrate that the tested compounds were differently recognized by these polyamine oxidases.

### *N*-alkylated and amide derivatives of *(R)*- and *(S)*-MeSpd as substrates of amine oxidases in rat liver homogenates

Hölttä [32] originally purified APAO from the rat liver which is a good source for the enzyme. There are not much data available about the tissue distribution of APAO and SMOX in animals or humans, but the available data show that liver has the second highest APAO activity among the 13 studied organs in rat [34,41,42]. APAO prefers the *N*^1^-Ac-*(R)*-MeSpd over to respective *(S)*-enantiomer [24]. The similar strong *(R)*-preference was true with bulky P4-, Bz- and Bn-MeSpd when rat liver supernatant was used as an enzyme source (Table 2). All the corresponding *(S)*-enantiomer derivatives were not degraded under the same experimental conditions. Complete inhibition of analogue degradation in the presence of MDL72527, an irreversible inhibitor of APAO and SMOX, clearly suggest that their degradation is mediated by APAO and/or SMOX. More importantly, human recombinant SMOX displayed very low *k*_cat_ and high *K*_i_ for the studied Spd derivatives (see above paragraph), thus clearly pointing to APAO as the degrading enzyme. These data indicate that *(S)*-1-methylation renders Spd analogue derivatives stable and could therefore be used to stabilize previously developed *N*-alkylated polyamine analogues for *in vivo* use. Furthermore, introduction of 1-methyl group could also alter biological response in comparison with parent compound [19,43].

### Substrate properties of Fms1 and the pH dependency of reaction using Spm as a substrate

Fms1 was originally characterized in yeast as a high-copy suppressor of the antifungal drug fenpropimorph. Its cloning and production as recombinant enzyme facilitated the characterization of its substrate specificity in 2003 [31]. The enzyme has been crystallized with several ligands and their structural data are available [16]. APAO, SMOX and Fms1 share many common features but their substrate specificities differ interestingly. SMOX prefers Spm over *N^1^*-AcSpm, and other polyamines or their acetylated derivatives are not substrates [40]. APAO prefers *N^1^-*AcSpm, *N^1^,N^12^-*DiAcSpm and *N^1^-*AcSpd while *N^8^-*AcSpd is an efficient inhibitor for the enzyme [29,44]. Fms1 cleaves at the exo-*N^4^*-site of *N^1^*-AcSpm > Spm > *N^1^*-AcSpd >> and endo-*N^4^*-site of *N^8^*-AcSpd [31]. Recent kinetic data of Fms1 by Adachi et al. [45] sets Spm (*k*_cat_ = 39.0 ± 1.5 s^−1^) > N^1^-AcSpm. (*k*_cat_ = 15.1 ± 0.4 s^−1^). APAO and SMOX cleave substrates at exo-*N^4^*-site, thus differentiating them from the maize PAO. Fms1 has the highest *k*_cat_ values for Spm in comparison with APAO or SMOX [25,29,31,45].

Here we used recombinant Fms1 having the activity of 30.9 ± 0.45 µmol/mg/min (*k*_cat_ = 30.3 ± 0.44 s^−1^) in Glycine-NaOH buffer at pH 9.0 and with 1 mM Spm as a substrate (Table 3). The reaction velocity was slightly enhanced in 100 mM Tris/HCl or 170 mM Bis/Tris buffers at pH 9.0 reaching 36.1 ± 0.24 µmol/mg/min. The use of HPLC for detecting reaction products allowed a reliable determination of reaction velocity compared with pH which could be hampered in peroxidase-coupled assay systems [30]. Reaction velocity was the highest at pH 9.25 and was retarded to 60% at pH 8.5 and to ~15% at pH 8.0 in comparison with reaction rate at the optimum pH (Supplementary Material 1, Figure S1). Determined pH dependency correlated with the data obtained by Adachi et al. [45]. The pH dependency of the reaction velocity was similar to that of APAO and SMOX [32,45–47]. The kinetic values of Fms1 for racemic Me_2_Spm, *(R,R)*-Me_2_Spm, *(S,S)*-Me_2_Spm and Spm are shown in Table 3. Despite 1,12-bis-methylation, the affinities of analogues for Fms1 were retained but the catalytic velocities dropped to less than one tenth in comparison with Spm. Thus, Fms1 tolerated 1,12-bis-methyl substituents in spite of their stereoconfiguration in Spm poorly in comparison with APAO and SMOX. In the case of APAO catalytic velocity using *(S,S)*-Me_2_Spm was slightly enhanced in comparison with Spm. Specificity constant ratios in using *(S,S)*-Me_2_Spm as a reference substrate between these polyamine oxidases are SMOX (SS/RR 454; SS/Spm 2.1)>>>APAO (SS/RR 28; SS/Spm 7.1)> Fms1 (SS/RR 3.9; SS/Spm 0.07) [25,29].

**Table 3:** Kinetic values for Spm and its 1,12-bis-methylated analogues as substrates of Fms1

Polyamine	*K*_m_ (μM)	*V*_max_ (μmol/min/mg)	*k*_cat_ (s^−1^)	*k*_cat_/*K*_m_ (M^−1^ s^−1^)
Spm^1^	77 ± 8	31.7 ± 1.0	31.1 ± 0.98	(400 ± 38) × 10^3^
Racemic Me_2_Spm^2^	54 ± 7	1.51 ± 0.05	1.48 ± 0.05	(27 ± 3.7) × 10^3^
*(R,R)*-Me_2_Spm^3^	98 ± 12	0.79 ± 0.03	0.77 ± 0.03	(7.9 ± 1.1) × 10^3^
*(S,S)*-Me_2_Spm^3^	61 ± 7	1.89 ± 0.05	1.85 ± 0.05	(30 ± 3.6) × 10^3^

Reactions were carried out in triplicates in 100 mM Glycine-NaOH buffer at pH 9.0 and analysed for reaction products as described in ‘Experimental procedures’ section. Turnover number (*k*_cat_) has been calculated by using *M*_r_ of 58833 for Fms1 monomer.

1 25, 50, 75, 100, 200, 400 and 600 µM concentrations were used.

2 25, 50, 100, 200, 400 and 1000 µM concentrations were used.

3 25, 50, 100, 200, 400, 600 and 1000 µM concentrations were used.

### Control of regioselectivity of Fms1 for Spd with aldehydes

Aldehyde supplementation has been successfully used to mimic *N*^1^-acetylation of Spd in APAO catalysis, since *N^1^-*AcSpd is a substrate of Fms1. We studied the effects of different aldehydes on substrate properties of Spd for Fms1 [29,31]. First, we found that Fms1 slowly degraded Spd and the *K*_m_ value for Spd was expectedly much higher than that for Spm and *N*^1^*-*AcSpd. The reaction was expected to yield Put and 3-aminopropanal, yet our HPLC analyses indicated that DAP was also produced (Table 4). This implies the presence of two cleavage sites, at exo- and at endo-*N*^4^-sites of Spd as reported earlier for *N*^1^- and *N*^8^-AcSpd respectively [31]. Table 4 shows the effects of various aldehydes (mimicking *N*^1^*-*AcSpd, *N*^8^*-*AcSpd and *N*^1^*,N*^8^-DiAcSpd) on the Fms1-catalysed reaction with Spd as the substrate. Unlike the human APAO reaction, where the aldehydes mainly increased *V*_max_ values, in the Fms1 reaction the aldehydes most profoundly decreased the *K*_m_ values. Table 4 also shows the two distinct cleavage sites, cleavage at E1 yielding Put and at E2 yielding DAP. In the absence of the aldehydes, the E1 route was strongly preferred. Most of the aldehydes enhanced the cleavage at E1, yet three of them (A6, A18 and A4) shifted the balance towards E2 cleavage site (Table 4). The aldehydes increased the ratio of the cleavage pathways (E1/E2) up to 5-fold (A7) and decreased it up to 12-fold (A4) at best. In most cases, the supplemented aldehydes brought about a dramatic increase in the enzyme efficiency (k_cat_/*K*_m_) at both cleavage sites. However, with the tested aldehydes the maximal reaction velocities of 1/10 of *k*_cat_ for E1 (*N*^1^*-*AcSpd) cleavage and approximately one-third for E2 (*N*^8^*-*AcSpd) cleavage were reached respectively. Thus, in the case of Fms1 using Spd as a substrate the supplementation of aromatic aldehydes to reaction mixture gives a possibility to control the regioselectivity of the reaction.

**Table 4:** Kinetic values of *N*^1^-AcSpd, *N*^8^-AcSpd, and Spd in the presence or absence of different aldehydes, for Fms1

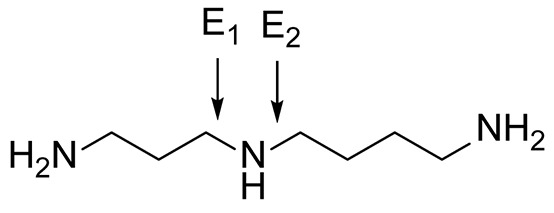		E_1_ cleavage kinetic values (Put)			E_2_ cleavage kinetic values (DAP)		
Substrate and/or supplementary aldehyde	Ratio of E_1_/E_2_	*K*_m_ (μM)	*k*_cat_ (s^−1^)	*k*_cat_/*K*_m_ (M^−1^s^−1^)	*K*_m_ (μM)	*k*_cat_ (s^−1^)	*k*_cat_/*K*_m_ (M^−1^s^−1^)
*N*^1^-AcSpd	NA	42 ± 8	65 ± 2	(1600 ± 300) × 10^3^	NA	NA	NA
*N*^8^-AcSpd	NA	NA	NA	NA	122 ± 18	1.4 ± 0.1	(12 ± 1.8) × 10^3^
Spd	7.5	534 ± 36	0.34 ± 0.01	640 ± 47	643 ± 52	0.05 ± 0.00	86 ± 7
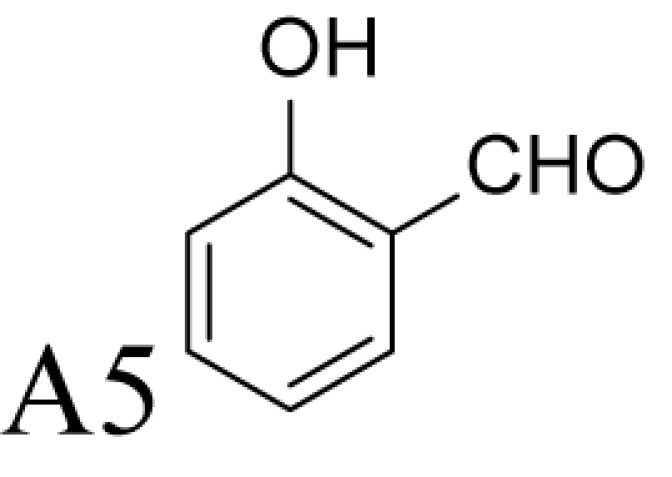	5.2	25 ± 3	0.54 ± 0.01	(22 ± 2.6) × 10^3^	18 ± 3	0.08 ± 0.00	(4.2 ± 0.8)×10^3^
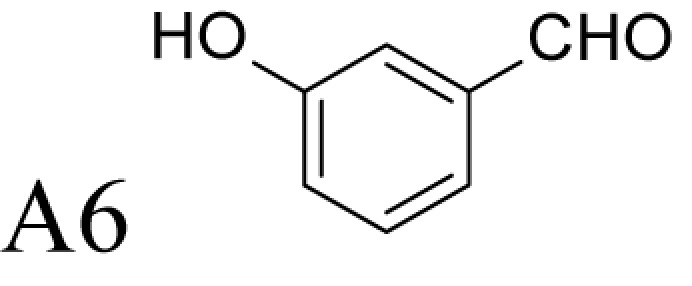	0.22	32 ± 3	0.31 ± 0.01	(0.97 ± 0.10) × 10^3^	107 ± 6	0.47 ± 0.01	(4.4 ± 0.3)×10^3^
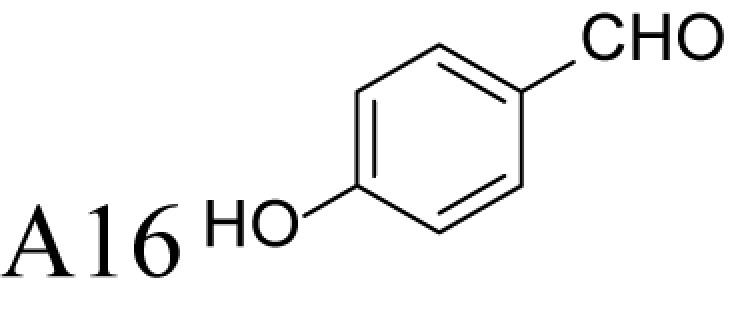	118	1.3 ± 1.0	0.11 ± 0.00	(85 ± 65) × 10^3^	42 ± 10	0.03 ± 0.00	(0.72 ± 0.17) × 10^3^
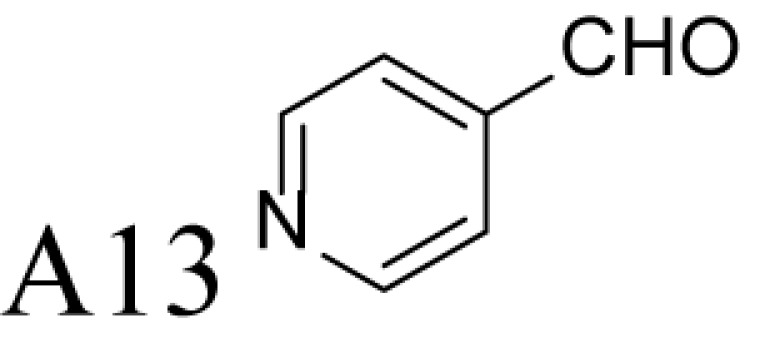	12.9	139 ± 9	7.4 ± 0.2	(53 ± 3.8) × 10^3^	47 ± 6	0.19 ± 0.01	(4.1 ± 0.6) × 10^3^
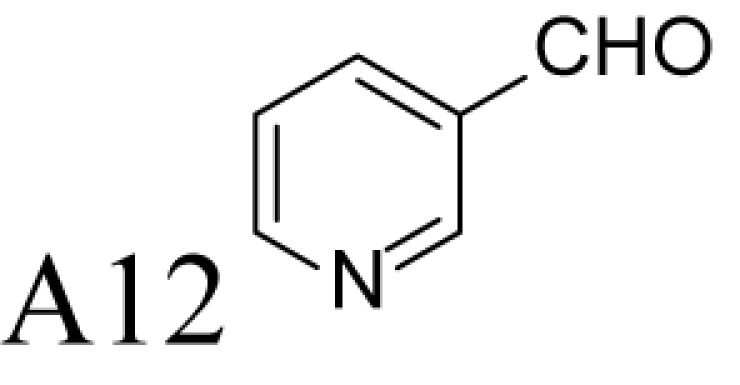	8.0	138 ± 8	5.5 ± 0.1	(40 ± 2.4) × 10^3^	96 ± 7	0.48 ± 0.01	(5.0 ± 0.4) × 10^3^
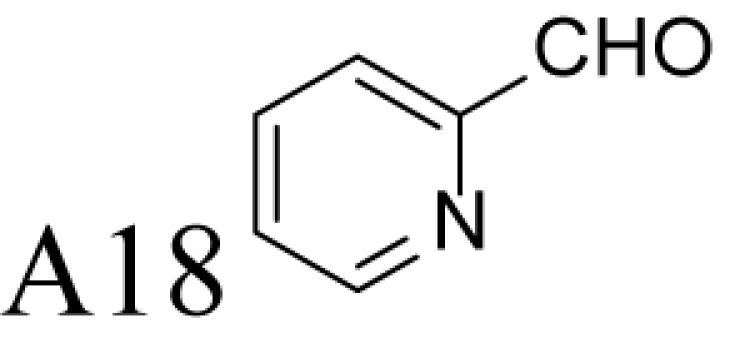	2.2	25 ± 2	0.49 ± 0.01	(19 ± 1.6) × 10^3^	55 ± 4	0.49 ± 0.01	(8.8 ± 0.7) × 10^3^
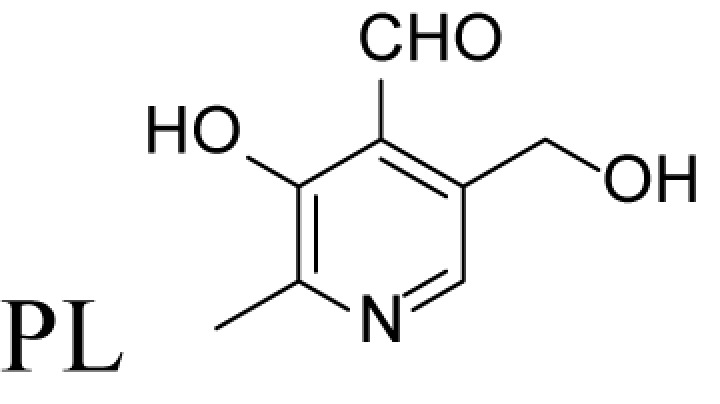	NA	33 ± 3	1.13 ± 0.03	(34 ± 3.2) × 10^3^	NA	NA	NA
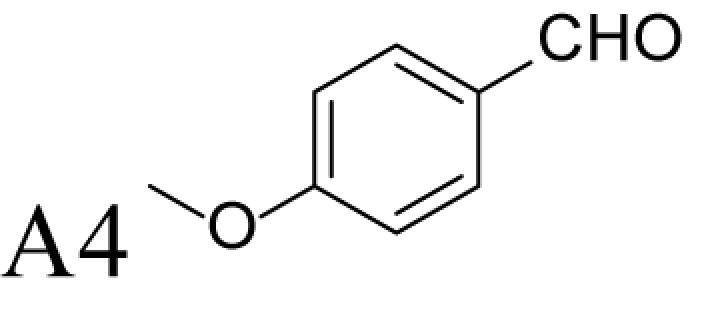	NA	NA	0.13 ± 0.00	NA	217 ± 10	0.22 ± 0.00	(1.0 ± 0.05) × 10^3^
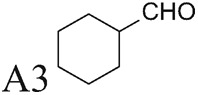	5.4	185 ± 16	1.41 ± 0.04	(7.6 ± 0.7) × 10^3^	296 ± 16	0.43 ± 0.01	(1.4 ± 0.09) × 10^3^
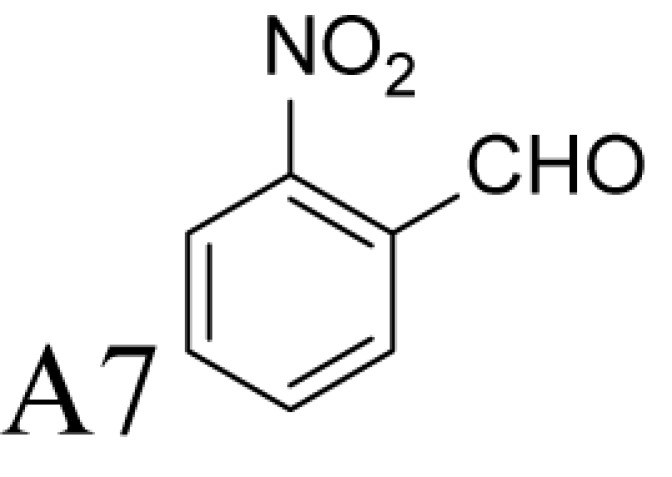	NA	16 ± 3	1.06 ± 0.04	(66 ± 13) × 10^3^	NA	0.03 ± 0.00	NA
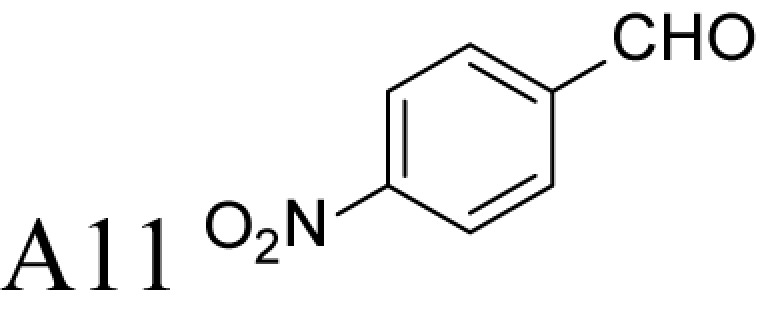	6.4	5.3 ± 0.9	0.37 ± 0.00	(70 ± 12) × 10^3^	4.7 ± 0.8	0.05 ± 0.00	(11 ± 1.8) × 10^3^

The reactions were carried out in triplicate at pH 9.0 in 100 mM Glycine-NaOH at +25°C with the fixed 1 mM Spd supplemented with increasing concentrations (25, 50, 75, 100, 250, 500 and 1000 μM) of tested aldehyde (Figure 2). Kinetic values for Spd were determined by using substrate concentrations of 50, 100, 200, 400, 600, 1000 and 4000 μM. Recombinant Fms1 was 1–2 μg/reaction and the incubation time from 5 to 30 min. Linearity of reaction was monitored by using T_1/2_ controls, i.e. samples that have been incubated for 2.5–15 min (half of the reaction time of an ordinary sample). *N*^1^AcSpd 50, 100, 300, 600 and 1000 µM 0.05 µg of Fms1 at 25°C 1 min. *N*^8^AcSpd 50, 100, 200 and 600 µM 0.59 µg of Fms1 at 25°C 10 min. Reaction mixtures without the enzyme supplement were used to control purity of the reagents and to exclude non-enzymatic degradation of the compounds. E_1_ cleavage was monitored by HPLC by measuring Put formation and E_2_ cleavage by determining DAP content. *k*_cat_ values have been calculated assuming *M*_r_ of 58833 for monomer with one catalytically active centre.

### *N*^1^-AcMeSpd and its *(R)-* and *(S)*-enantiomers as substrates of Fms1

The human recombinant APAO readily catalysed oxidation of *N^1^*-Ac-(*R*)-MeSpd and Schiff bases of MeSpd with aromatic aldehydes [24,29]. Unexpectedly, Fms1 did not metabolize neither of *(R)-* and *(S)*-enantiomers of *N*^1^-Ac-MeSpds (Supplementary Material 1, Table S1). Accordingly, *(R)*- and *(S)*-MeSpd had similar (*K*_m_ > 500 µM) as Spd (Supplementary Material 1, Table S2). Above applies to both of the tested aldehydes A12 and A13 (50 and 500 µM) with 1 or 4 mM *(R-)* or *(S)*-MeSpd (Supplementary Material 1, Table S3).

## Conclusion

The obtained data clearly demonstrate that Fms1 and APAO (both using achiral natural polyamines as substrates) appear to be representative examples of enzymes, whose stereospecificity and regioselectivity can be modulated by small guide molecules. Having established that Spd in Fms1 reaction has two cleavage sites, i.e. exo-*N^4^*-site (E_1_) and endo-*N^4^*-site (E_2_), it turned out to be possible to induce predominant cleavage at either (E_1_) or (E_2_) site by minor changes of the structure of supplemented aromatic aldehyde needed to form ***in situ*** a novel substrate—Schiff base with Spd. The same ‘aldehyde approach’ in the case of APAO and chiral 1-MeSpds’ provided a unique possibility to induce cleavage of either *(R)*- or *(S)*-isomer depending on the structure of used aromatic aldehyde. Fms1 like APAO exhibits hidden stereospecificity and prefers *(S,S)*- to *(R,R)*-Me_2_Spm diastereoisomer with notably lower *k*_cat_ in comparison with Spm. The present data together with earlier accumulated knowledge of polyamine analogue structure–bioactivity relationships allow deriving novel chemico-biological applications to modulate cell physiology and generation of specific substrates or inhibitors for polyamine metabolizing enzymes.

## Supporting information

**Supplementary Figure 1:** Catalytic velocities of Fms1 with 1 mM Spm as a substrate in 170 mM Bis-Tris buffer at different pH

**Supplementary Table 1:** N^1^-AcSpd and N^1^-Acetylated MeSpd as substrates of Fms1

**Supplementary Table 2:** Enantiomers of MeSpd as substrates of Fms1.

**Supplementary Table 3:** The effects of increasing aromatic aldehyde concentration for the regioselectivity of Fms1 using (*R*)-MeSpd or (*S*)-MeSpd as a substrate.

## Abbreviations

APAO: acetylpolyamine oxidase
DAP: 1,3-diaminopropane
DHS: deoxyhypusine synthase
Fms1: yeast polyamine oxidase
MeSpd: 1-methylspermidine (1,8-diamino-5-azanonane)
Me_2_Spm: 1,12-bis-methylspermine (2,13-diamino-5,10-diazatetradecane)
Put: putrescine
SMOX: spermine oxidase
Spd: spermidine
Spm: spermine
SSAT: Spd/Spm-*N^1^*-acetyltransferase
