# Supplementary material for: Controlling the regioselectivity and stereospecificity of FAD-dependent polyamine oxidases with the use of amine-attached guide molecules as conformational modulators
Source: Biosci Rep. 2018 Aug 29;38(4):BSR20180527. doi: 10.1042/BSR20180527 (PMC6131205; doi:10.1042/BSR20180527)
Supplement: Supplementary file 1 [file bsr20180527_Supp1.pdf]

Supplementary Material 1

Controlling the regioselectivity and stereospecificity of FAD-dependent polyamine oxidases with the use of amine-attached guide molecules as conformational modulators

Tuomo A. Keinänen*[,1], Nikolay Grigorenko[‡], Alex R. Khomutov[§], Qingqiu Huang[†], Anne Uimari[‖], Leena Alhonen*, Mervi T. Hyvönen* and Jouko Vepsäläinen*

**School of Pharmacy, Biocenter Kuopio, University of Eastern Finland, Kuopio Campus, P.O. Box 1627 Kuopio, FI-70211 Finland. [‖]Natural Resources Institute Finland, Neulaniementie 5, FI-70210 Kuopio, Finland. [†]MacCHESS at the Cornell High Energy Synchrotron Source, Cornell University Ithaca, NY 14853-8001, USA. [‡]BASF Schweiz AG, P.O. Box, CH 4002, Basel, Switzerland. [§]Engelhardt Institute of Molecular Biology, Russian Academy of Sciences, Vavilov St 32, 119991 Moscow, Russia.*

[1] Corresponding author Tuomo.Keinanen@uef.fi

Abbreviations:

Fms1, yeast polyamine oxidase; MeSpd, 1-methylspermidine (1,8-diamino-5-azanonane); Me_2Spm, 1,12-dimethylspermine (2,13-Diamino-5,10-diazatetradecane); N^1-Ac-MeSpd, N^8-Acetyl-1,8-diamino-5-azanonane.

Supplementary Table 1. N^1-AcSpd and N^1-Acetylated MeSpd as substrates of Fms1.

The reactions were carried out in triplicate at pH 9.0 in 100 mM Glycine-NaOH at +25°C. Kinetic values for N^1-Ac-MeSpd enantiomers were determined by using substrate concentrations of 25, 50, 100, 200, 400, 600 and 1000 μM. Recombinant Fms1 was 1.8 μg/reaction and the incubation times for Rac-MeSpd 30 min, (*R*)-MeSpd 20 min and (*S*)-MeSpd 40 minutes. Linearity of reaction was monitored by using $T_{1/2}$ controls, i.e. samples that have been incubated for 15, 10 and 20 min, respectively (half of the reaction time of an ordinary sample). Put formation was monitored by using HPLC. k_{cat} values have been calculated assuming M_r of 58,833 for monomer with one catalytically active centre.

**Polyamine**	**K_m (μM)**	**V_{max} (nmol/min/mg)**	**k_{cat} s^{-1}**	**k_{cat}/K_m $M^{-1}s^{-1}$**
N^1-AcSpd	42 ± 8	66,600 ± 2,300	65 ± 2	(1.56 ± 0.30) ×10^6
N^1-Ac-MeSpd	140 ± 23	50 ± 3	0.05 ± 0.00	357 ± 61
N^1-Ac-*(R)*-MeSpd	155 ± 29	61 ± 4	0.06 ± 0.00	387 ± 76
N^1-Ac-*(S)*-MeSpd	109 ± 18	22 ± 1	0.02 ± 0.00	183 ± 32

Supplementary Table 2 Enantiomers of MeSpd as substrates of Fms1.

The reactions were carried out in triplicate at pH 9.0 in 100 mM Glycine-NaOH at +25°C. Kinetic values for MeSpd enantiomers were determined by using substrate concentrations of 100, 300, 600, 1000, 2000 and 4000 μM. Recombinant Fms1 was 3.8 μg/reaction and the incubation time 60 minutes. Linearity of reaction was monitored by using $T_{1/2}$ controls, i.e. samples that have been incubated for 30 min (half of the reaction time of an ordinary sample). E_1 cleavage was monitored by HPLC by measuring Put formation and E_2 cleavage by determining butane-1,3-diamine content. k_{cat} values have been calculated assuming M_r of 58,833 for monomer with one catalytically active centre.

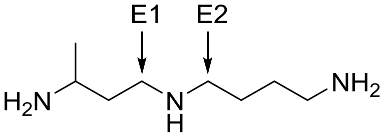	**E_1 cleavage kinetic values**			**E_2 cleavage kinetic values**		
Substrate	K_m (μM)	k_{cat} s^{-1}	k_{cat}/ K_m	K_m (μM)	k_{cat} s^{-1}	k_{cat}/ K_m
(R)-MeSpd	502 ± 31	0.014 ± 0	28 ± 2	467 ± 87	0.006 ± 0	13 ± 3
(S)-MeSpd	555 ± 33	0.011 ± 0	20 ± 1	606 ± 99	0.013 ± 0	22 ± 4

Supplementary Table 3 The effects of increasing aromatic aldehyde concentration for the regioselectivity of Fms1 using *(R)*-MeSpd or *(S)*-MeSpd as a substrate.

The reactions were carried out in triplicate at pH 9.0 in 100 mM Glycine-NaOH at +25°C with the fixed 1 or 4 mM MeSpd supplemented with increasing concentration 50 or 500 µM of tested aldehyde. Recombinant Fms1 was 1.6 µg/reaction and the incubation time from 15 to 30 minutes. Linearity of reaction was monitored by using $T_{1/2}$ controls, i.e. samples that have been incubated for 7.5 to 15 min (half of the reaction time of an ordinary sample). E_1 cleavage was monitored by HPLC by measuring Put formation and E_2 cleavage by determining butane-1,3-diamine content.

Substrate		*(R)*-MeSpd		*(S)*-MeSpd	
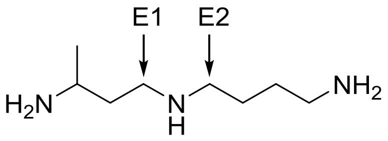		E1 (*nmol/mg/min)*	E2 (*nmol/mg/min)*	E1 (*nmol/mg/min)*	E2 (*nmol/mg/min)*
Without aldehyde 1 mM		9.5 ± 0.1	4.3 ± 0.9	7.2 ± 0.2	7.9 ± 1.1
Without aldehyde **4 mM**		**12.5 ± 0.0**	**5.6 ± 0.4**	**9.6 ± 0.1**	**11.1 ± 1.8**
aldehyde	*(µM)*				
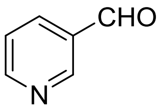 A12	50	10.5 ± 1.1	33.7 ± 4.8	29 ± 1.0	20.2 ± 1.0
	**50**	14.0 ± 0.9	31.8 ± 4.3	31 ± 1.8	23.3 ± 2.0
	500	12.7 ± 1.6	186 ± 24	57 ± 1.1	36 ± 4.2
	**500**	14.4 ± 0.8	216 ± 31	62 ± 3	41 ± 2.5
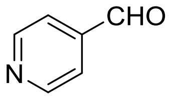 A13	50	19.1 ± 1.0	10.3 ± 3.1	45.6 ± 2.6	8.6 ± 1.8
	**50**	19.1 ± 0.4	9.6 ± 2.2	44.0 ± 1.2	10.0 ± 0.6
	500	72.8 ± 2.3	41.5 ± 3.4	66.1 ± 0.0	6.9 ± 0.7
	**500**	63.1 ± 1.0	39.5 ± 2.1	73.5 ± 0.4	7.4 ± 1.3

Supplementary Figure 1

Catalytic velocities of Fms1 with 1 mM Spm as a substrate in 170 mM Bis-Tris buffer at different pH

200 mM (170 mM in the final reaction mixtures) Bis-Tris propane buffer was prepared at pH 7.4; 8.0; 8.5; 8.8; 9.0; 9.2 and 9.5. Fms1 0.1 µg/reaction mixture was supplemented with 1 mM Spm at various pH V_{tot} 180 µl. Reaction mixtures were incubated at 25 °C water bath for four minutes until 20 µl of 50 % w/v SSA containing 100 uM DAH was added and vortexed briefly and placed on ice. HPLC was used to analyse Spd content of samples as described in Experimental Procedures. Values are averages of triplicate determinations expressed as µmol/min ± SD (error bars).

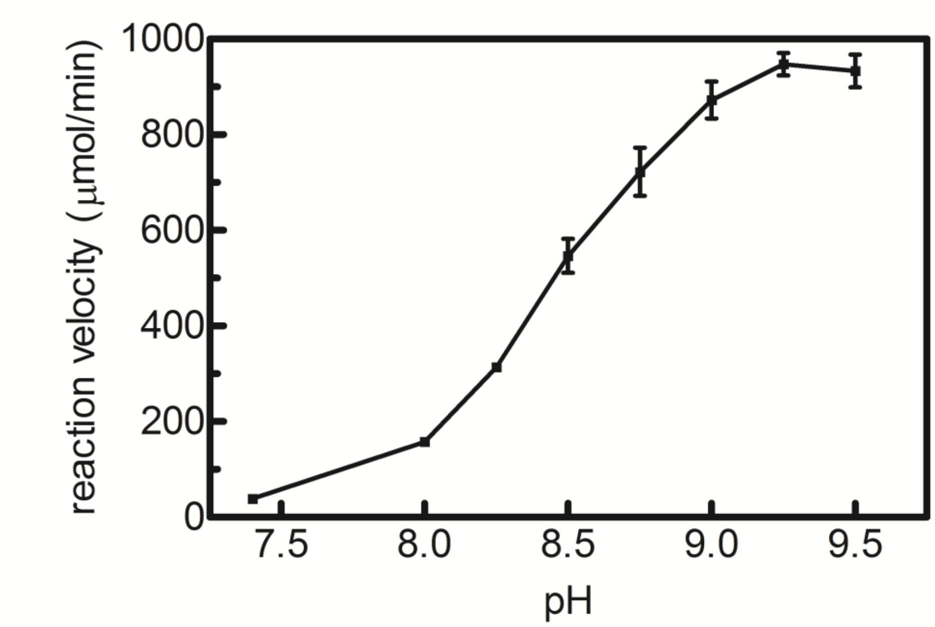

Supplementary Material 2

Controlling the regioselectivity and stereospecificity of FAD-dependent polyamine oxidases with the use of amine-attached guide molecules as conformational modulators

Tuomo A. Keinänen*[,1], Nikolay Grigorenko[‡], Alex R. Khomutov[§], Qingqiu Huang[†], Anne Uimari[‖], Leena Alhonen*, Mervi T. Hyvönen* and Jouko Vepsäläinen*

**School of Pharmacy, Biocenter Kuopio, University of Eastern Finland, Kuopio Campus, P.O. Box 1627 Kuopio, FI-70211 Finland. [‖]Natural Resources Institute Finland, Neulaniementie 5, FI-70210 Kuopio, Finland. [†]MacCHESS at the Cornell High Energy Synchrotron Source, Cornell University Ithaca, NY 14853-8001, USA. [‡]BASF Schweiz AG, P.O. Box, CH 4002, Basel, Switzerland. [§]Engelhardt Institute of Molecular Biology, Russian Academy of Sciences, Vavilov St 32, 119991 Moscow, Russia.*

[1] Corresponding author Tuomo.Keinanen@uef.fi

Michaelis-Menten Graphs and Data of Table 1

GraphPad Prism 5.03	N^1AcSpd	Bn-R-MeSpd	Bn-S-MeSpd	Bz-R-MeSpd	Bz-S-MeSpd	P4-R-MeSpd	P4-S-MeSpd
	µmol/min/mg	µmol/min/mg	µmol/min/mg	µmol/min/mg	µmol/min/mg	µmol/min/mg	µmol/min/mg
Michaelis-Menten							
Best-fit values							
V_{max}	2.969	0.1534	0.0307	5.017	0.02985	5.593	0.01522
K_m	8.218	2.039	1.639	1.214	0.2098	3.645	0.839
Std. Error							
V_{max}	0.02437	0.001549	0.001783	0.5122	0.000935	0.2399	0.0003134
K_m	0.3908	0.109	0.591	0.7426	0.161	0.5973	0.1498
95% Confidence Intervals							
V_{max}	2.901 to 3.037	0.1467 to 0.1600	0.02303 to 0.03838	3.387 to 6.647	0.02687 to 0.03282	4.928 to 6.259	0.01435 to 0.01609
K_m	7.133 to 9.303	1.570 to 2.508	0.0 to 4.182	0.0 to 3.577	0.0 to 0.7221	1.987 to 5.303	0.4233 to 1.255
Goodness of Fit							
Degrees of Freedom	4	2	2	3	3	4	4
R square	0.9962	0.9962	0.8491	0.6415	0.385	0.9614	0.9165
Absolute Sum of Squares	0.003966	0.000002157	0.000003144	0.7509	0.000003943	0.2719	0.0000007842
Sy.x	0.03149	0.001038	0.001254	0.5003	0.001146	0.2607	0.0004428
Constraints							
K_m	Km > 0.0	Km > 0.0	Km > 0.0	Km > 0.0	Km > 0.0	Km > 0.0	Km > 0.0
Number of points							
Analyzed	6	4	4	5	5	6	6

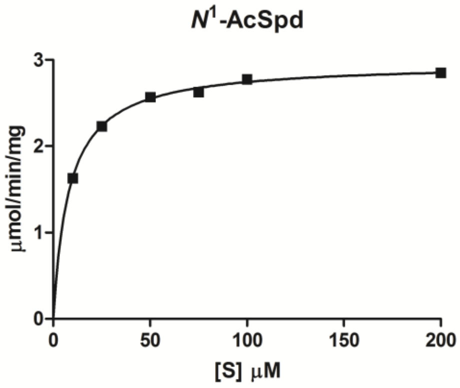
N^1-AcSpd
μmol/min/mg
[S] μM

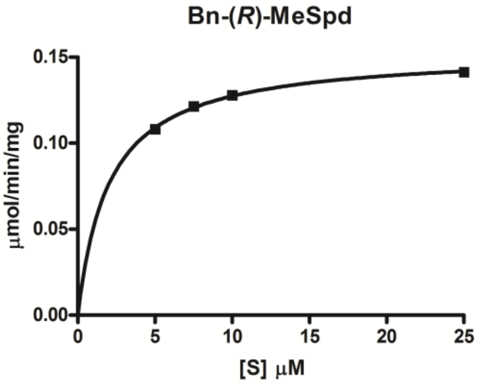
Bn-(*R*)-MeSpd
μmol/min/mg
[S] μM

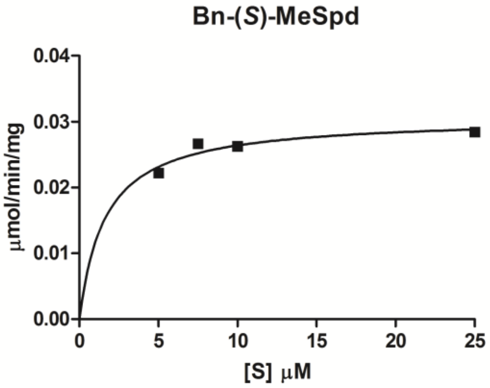
Bn-(*S*)-MeSpd
μmol/min/mg
[S] μM

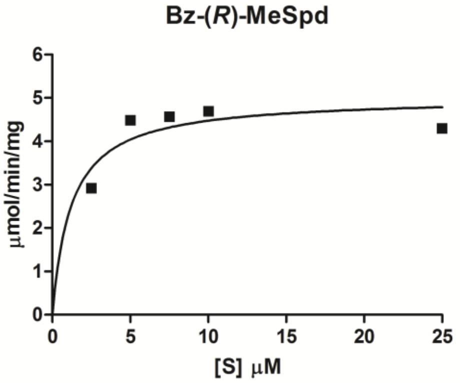
Bz-(*R*)-MeSpd
μmol/min/mg
[S] μM

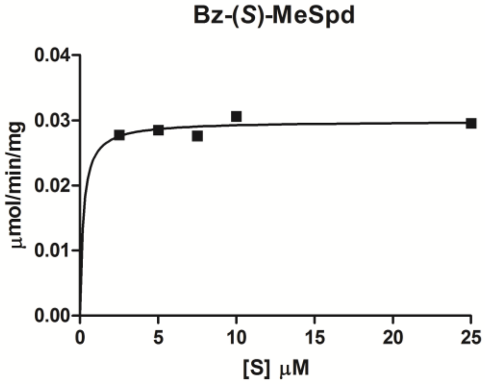
Bz-(*S*)-MeSpd
μmol/min/mg
[S] μM

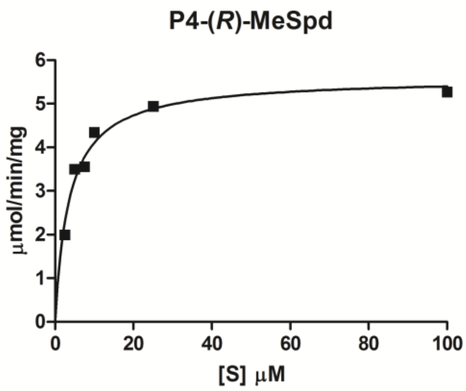
P4-(*R*)-MeSpd
μmol/min/mg
[S] μM

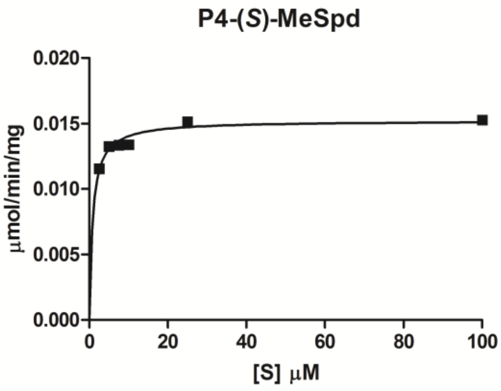
P4-(*S*)-MeSpd
μmol/min/mg
[S] μM

Michaelis-Menten Graphs and Data of Table 3

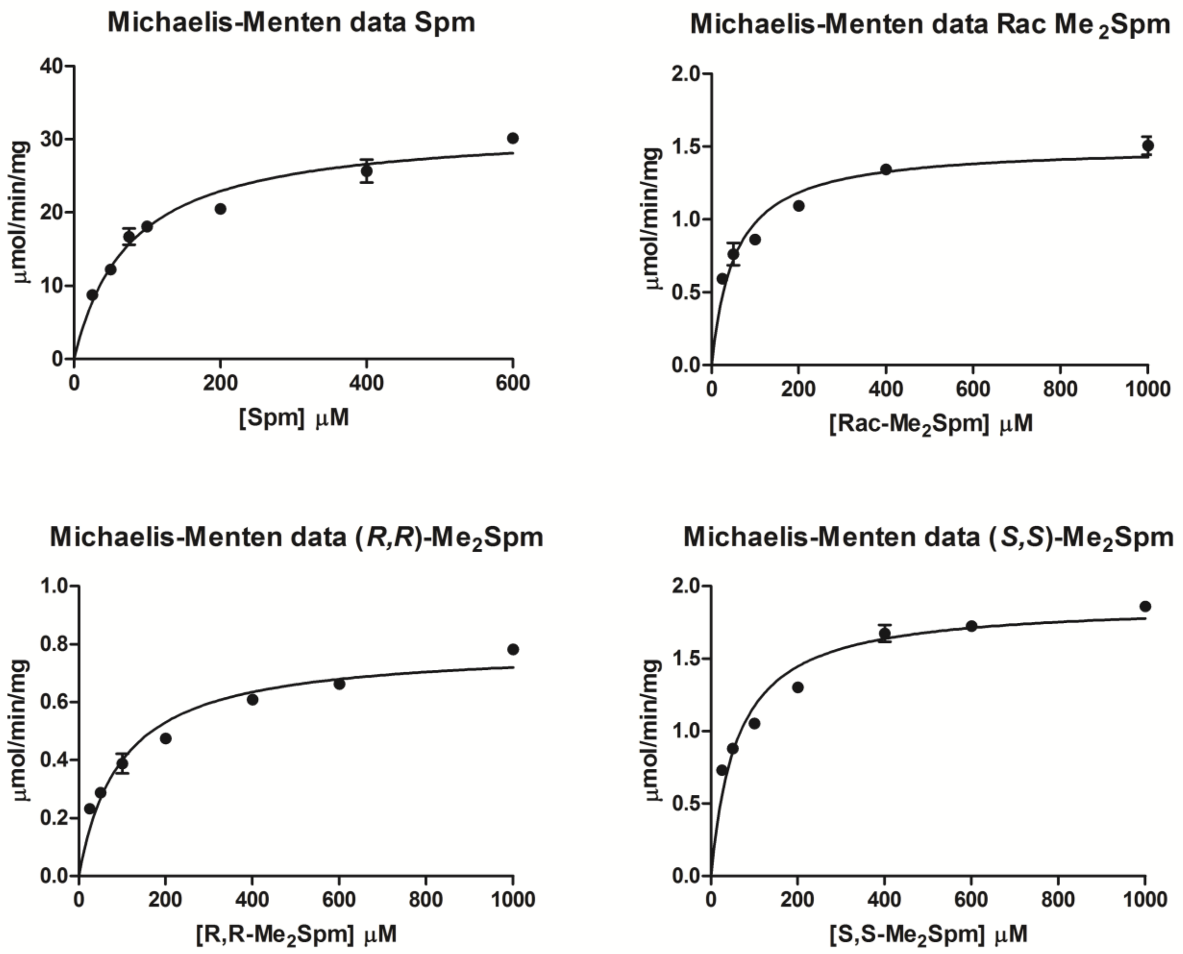

GraphPad Prism 5.03	Spm	Rac-Me$_2$Spm	(*R,R*)-Me$_2$Spm	(*S,S*)-Me$_2$Spm
	μmol/min/mg	μmol/min/mg	μmol/min/mg	μmol/min/mg
Michaelis-Menten				
Best-fit values				
V_{max}	31.71	1.506	0.7903	1.886
K_m	76.85	54.4	97.9	60.81
Std. Error				
V_{max}	1.030	0.05122	0.02751	0.05232
Km	7.842	7.168	12.37	7.038
95% Confidence Intervals				
V_{max}	29.55 to 33.86	1.397 to 1.614	0.7327 to 0.8479	1.777 to 1.996
K_m	60.43 to 93.26	39.20 to 69.60	72.02 to 123.8	46.08 to 75.54
Goodness of Fit				
Degrees of Freedom	19	16	19	19
R square	0.9511	0.9181	0.9392	0.9321
Absolute Sum of Squares	48.65	0.1543	0.04538	0.2437
Sy.x	1.600	0.09819	0.04887	0.1133
Constraints				
K_m	Km > 0.0	Km > 0.0	Km > 0.0	Km > 0.0
Number of points				
Analyzed	21	18	21	21

Michaelis-Menten Graphs and Data of Table 4 (N^1-AcSpd data shown in Supplementary 1 Table1)

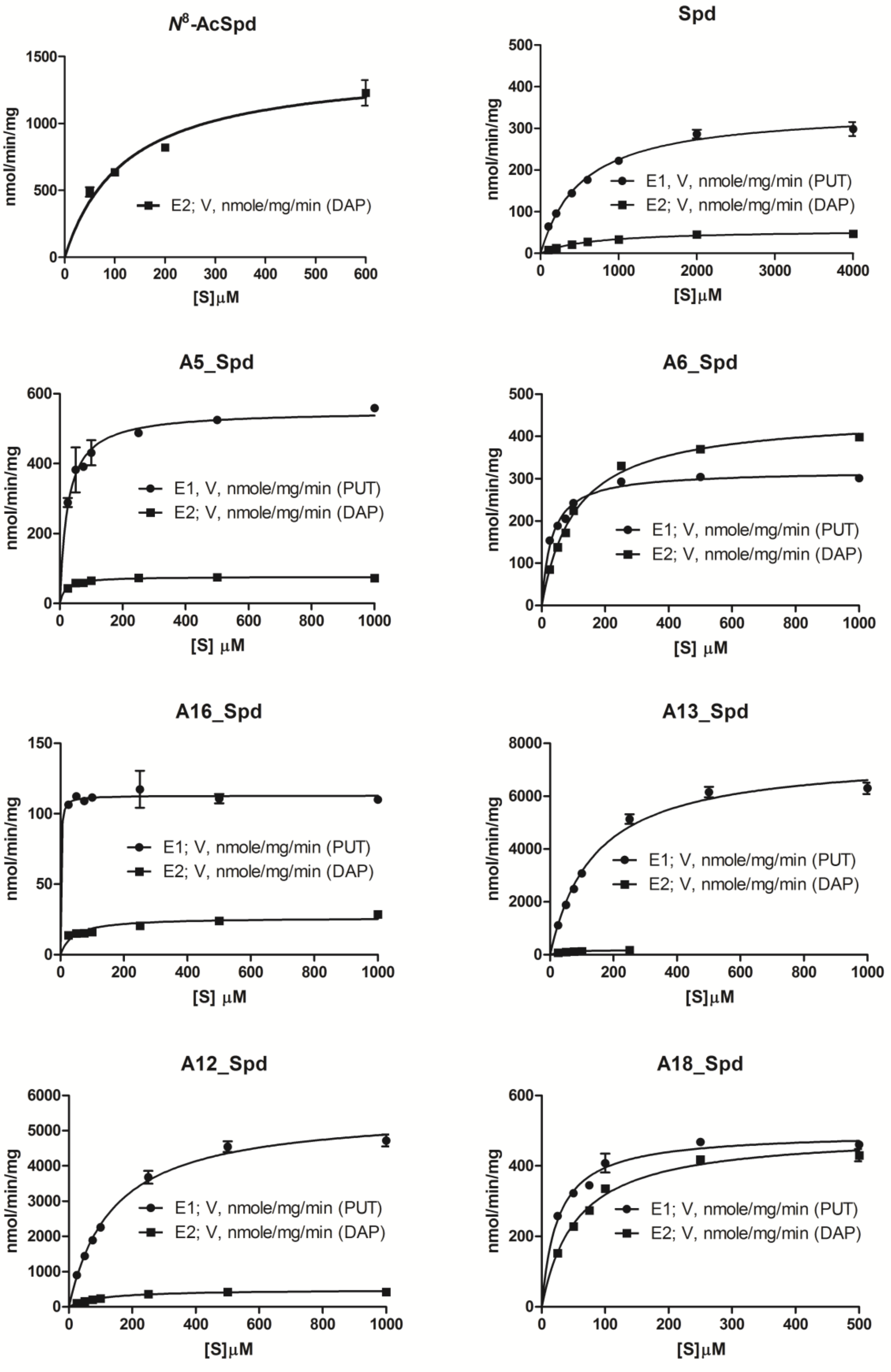

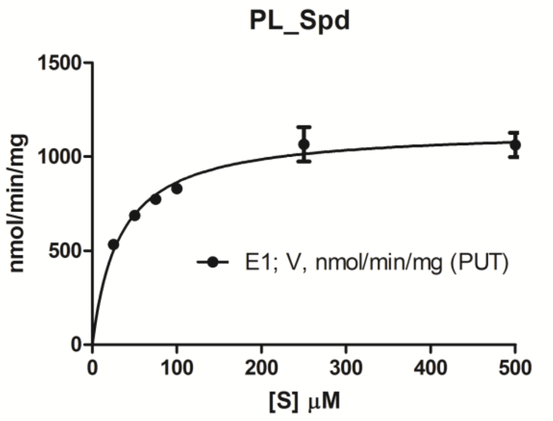
PL_Spd
1500
1000
500
0
nmol/min/mg
0
100
200
300
400
500
[S] μM
E1; V, nmol/min/mg (PUT)

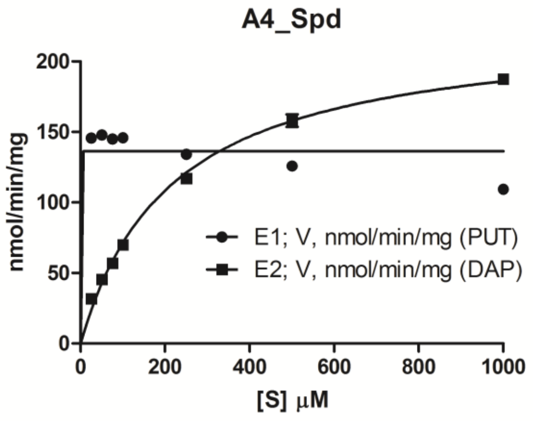
A4_Spd
200
150
100
50
0
nmol/min/mg
0
200
400
600
800
1000
[S] μM
E1; V, nmol/min/mg (PUT)
E2; V, nmol/min/mg (DAP)

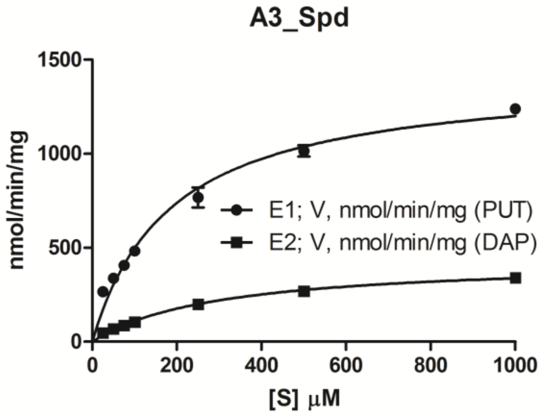
A3_Spd
1500
1000
500
0
nmol/min/mg
0
200
400
600
800
1000
[S] μM
E1; V, nmol/min/mg (PUT)
E2; V, nmol/min/mg (DAP)

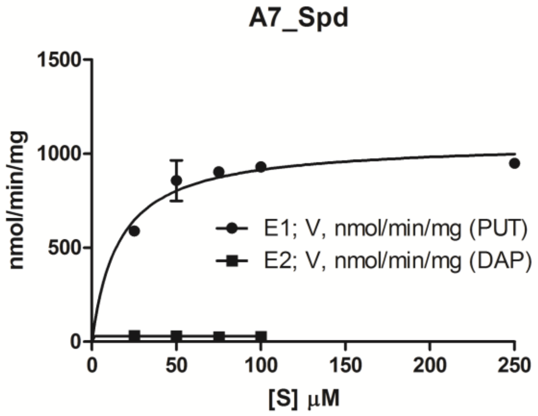
A7_Spd
1500
1000
500
0
nmol/min/mg
0
50
100
150
200
250
[S] μM
E1; V, nmol/min/mg (PUT)
E2; V, nmol/min/mg (DAP)

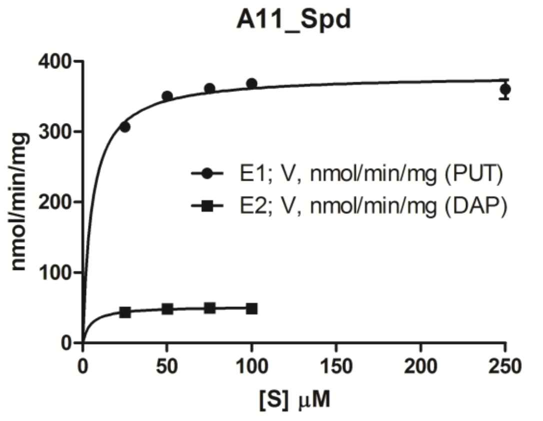
A11_Spd
400
300
200
100
0
nmol/min/mg
0
50
100
150
200
250
[S] μM
E1; V, nmol/min/mg (PUT)
E2; V, nmol/min/mg (DAP)

GraphPad Prism 5.03	N^8AcSpd	Spd E1	Spd E2	A5_Spd E1	A5_Spd E2	A6_Spd E1	A6_Spd E2
	nmol/min/mg	nmol/min/mg	nmol/min/mg	nmol/min/mg	nmol/min/mg	nmol/min/mg	nmol/min/mg
Michaelis-Menten					E1	E2	E1
Best-fit values							
V_{max}	1438	345.8	56.29	550.5	76.72	318.1	450.0
K_m	122.5	533.9	642.8	24.87	17.86	32.06	107.2
Std. Error							
V_{max}	78.14	7.799	1.627	13.54	1.878	5.977	7.656
K_m	18.36	35.89	52.36	3.075	2.595	2.710	5.632
95% Confidence Intervals							
V_{max}	1264 to 1612	329.5 to 362.2	52.88 to 59.69	521.1 to 580.0	72.63 to 80.81	305.1 to 331.1	433.3 to 466.6
K_m	81.60 to 163.4	458.8 to 609.0	533.2 to 752.4	18.17 to 31.57	12.20 to 23.51	26.16 to 37.97	94.89 to 119.4
Goodness of Fit							
Degrees of Freedom	10	19	19	12	12	12	12
R square	0.9404	0.9846	0.9804	0.9191	0.8833	0.9647	0.9933
Absolute Sum of Squares	56423	2276	81.23	8893	192.4	1562	1205
Sy.x	75.12	10.95	2.068	27.22	4.004	11.41	10.02
Constraints							
K_m	Km > 0,0	Km > 0.0	Km > 0.0	Km > 0.0	Km > 0.0	Km > 0.0	Km > 0.0
Number of points							
Analyzed	12	21	21	14	14	14	14

GraphPad Prism 5.03	A16_Spd E1	A16_Spd E2	A13_Spd E1	A13_Spd E2	A12_Spd E1	A12_Spd E2	A18_Spd E1	A18_Spd E2
	nmol/min/mg	nmol/min/mg	nmol/min/mg	nmol/min/mg	nmol/min/mg	nmol/min/mg	nmol/min/mg	nmol/min/mg
Michaelis-Menten	E2			E1	E2	E1	E2	
Best-fit values								
V_{max}	112.9	26.35	7527	198.6	5562	489.6	495.2	492.9
K_m	1.317	41.54	138.9	46.79	138.0	95.59	25.38	54.86
Std. Error								
V_{max}	1.894	1.490	171.8	9.048	105.8	11.4	10.63	10.19
K_m	0.9901	9.555	9.217	6.131	7.645	7.065	2.419	3.630
95% Confidence Intervals								
V_{max}	108.8 to 117.0	23.10 to 29.60	7167 to 7887	179.1 to 218.2	5341 to 5783	465.8 to 513.5	472.7 to 517.8	471.3 to 514.5
K_m	0.0 to 3.475	20.72 to 62.36	119.6 to 158.1	33.55 to 60.03	122.0 to 154.0	80.80 to 110.4	20.25 to 30.51	47.16 to 62.55
Goodness of Fit								
Degrees of Freedom	12	12	19	13	19	19	16	16
R square	0.1380	0.7713	0.9860	0.9309	0.9899	0.9772	0.9374	0.9797
Absolute Sum of Squares	285.7	86.03	1.120e+006	1075	427845	6996	6620	3649
Sy.x	4.879	2.677	242.8	9.094	150.1	19.19	20.34	15.10
Constraints								
K_m	Km > 0.0	Km > 0.0	Km > 0.0	Km > 0.0	Km > 0.0	Km > 0.0	Km > 0.0	Km > 0.0
Number of points								
Analyzed	14	14	21	15	21	21	18	18

GraphPad Prism 5.03	PL_Spd E1	A4_Spd E1	A4_Spd E2	A3_Spd E1	A3_Spd E2	A7_Spd E1	A7_Spd E2	A11_Spd E1	A11_Spd E2
	nmol/min/mg	nmol/min/mg	nmol/min/mg	nmol/min/mg	nmol/min/mg	nmol/min/mg	nmol/min/mg	nmol/min/mg	nmol/min/mg
Michaelis-Menten					E1	E2	E1	E2	
Best-fit values									
V_{max}	1148	136.3	226.3	1422	438.5	1062	29.53	380.9	52.45
K_m	32.75	~ 1.284e-016	216.6	184.7	295.7	16.08	~ 1.883e-016	5.338	4.719
Std. Error									
V_{max}	29.82	4.365	3.984	44.78	9.985	41.33	1.964	5.627	0.8756
K_m	3.352		9.974	15.82	16.23	3.137		0.8642	0.8453
95% Confidence Intervals									
V_{max}	1084 to 1211	127.2 to 145.5	218.0 to 234.7	1328 to 1515	417.6 to 459.4	972.5 to 1151	25.16 to 33.91	368.7 to 393.0	50.50 to 54.40
K_m	25.65 to 39.86		195.8 to 237.5	151.6 to 217.8	261.7 to 329.7	9.307 to 22.86		3.471 to 7.205	2.836 to 6.603
Goodness of Fit									
Degrees of Freedom	16	19	19	19	19	13	10	13	10
R square	0.9349	-4,441e-016	0.9946	0.9780	0.9940	0.8107	-4.441e-016	0.7998	0.8047
Absolute Sum of Squares	44961	3746	349.7	54639	1370	54501	110.0	1586	15.59
Sy.x	53.01	14.04	4.290	53.63	8.491	64.75	3.317	11.04	1.249
Constraints									
K_m	Km > 0.0	Km > 0.0	Km > 0.0	Km > 0.0	Km > 0.0	Km > 0.0	Km > 0.0	Km > 0.0	Km > 0.0
Number of points									
Analyzed	18	21	21	21	21	15	12	15	12

Inhibition of SMO by covalently modified (*R*)-MeSpd (*S*)-MeSpd derivatives

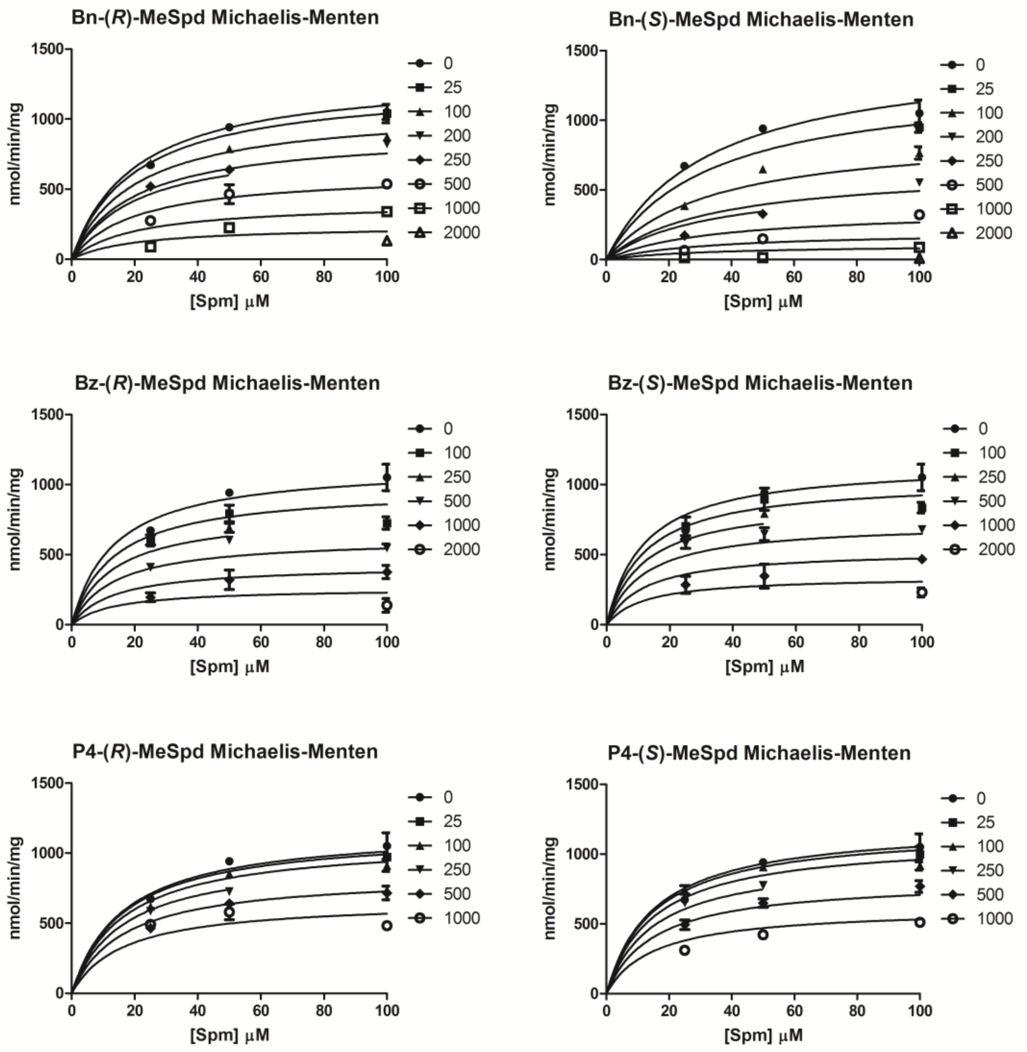

GraphPad Prism 5.03	Global (shared)	Global (shared)	Global (shared)	Global (shared)	Global (shared)	Global (shared)
Noncompetitive inhibition	Bn-(*R*)-MeSpd	Bn-(*S*)-MeSpd	Bz-(*R*)-MeSpd	Bz-(*S*)-MeSpd	P4-(*R*)-MeSpd	P4-(*S*)-MeSpd
Best-fit values						
V_{max}	1320	1502	1141	1154	1178	1215
I						
K_i	440.8	155	589	846	1277	1016
K_m	20.26	32.94	13.41	11.79	16.45	15.21
Std. Error						
V_{max}	50.8	89.27	57.86	53.07	36.78	36.77
K_i	32.26	12.61	56.77	81.95	110.7	79.12
K_m	2.792	5.471	2.831	2.422	2.009	1.900
95% Confidence Intervals						
V_{max}	1218 to 1422	1322 to 1681	1024 to 1258	1047 to 1261	1104 to 1252	1140 to 1289
K_i	375.9 to 505.7	129.6 to 180.3	474.4 to 703.6	680.6 to 1011	1054 to 1500	856.4 to 1176
K_m	14.65 to 25.88	21.93 to 43.95	7.695 to 19.13	6.904 to 16.68	12.40 to 20.51	11.38 to 19.05
Goodness of Fit						
Degrees of Freedom	48	48	42	42	42	42
R square	0.9526	0.9594	0.907	0.893	0.9071	0.9299
Absolute Sum of Squares	216629	255707	264780	267496	144692	146945
Sy.x	67.18	72.99	79.4	79.81	58.69	59.15

Bn-(*R*)-MeSpd

	Nonlin fit	A	B	C	D	E	F	G	H	I
		0	25	100	200	250	500	1000	2000	Global (shared)
		Y	Y	Y	Y	Y	Y	Y	Y	Y
1	Noncompetitive inhibition									
2	Best-fit values									
3	Vmax	1320	1320	1320	1320	1320	1320	1320	1320	1320
4	I	= 0.0	= 25.00	= 100.0	= 200.0	= 250.0	= 500.0	= 1000	= 2000	
5	Ki	440.8	440.8	440.8	440.8	440.8	440.8	440.8	440.8	440.8
6	KM	20.26	20.26	20.26	20.26	20.26	20.26	20.26	20.26	20.26
7	Std. Error									
8	Vmax	50.80	50.80	50.80	50.80	50.80	50.80	50.80	50.80	50.80
9	Ki	32.26	32.26	32.26	32.26	32.26	32.26	32.26	32.26	32.26
10	KM	2.792	2.792	2.792	2.792	2.792	2.792	2.792	2.792	2.792
11	95% Confidence Intervals									
12	Vmax	1218 to 1422	1218 to 1422	1218 to 1422	1218 to 1422	1218 to 1422	1218 to 1422	1218 to 1422	1218 to 1422	1218 to 1422
13	Ki	375.9 to 505.7	375.9 to 505.7	375.9 to 505.7	375.9 to 505.7	375.9 to 505.7	375.9 to 505.7	375.9 to 505.7	375.9 to 505.7	375.9 to 505.7
14	KM	14.65 to 25.88	14.65 to 25.88	14.65 to 25.88	14.65 to 25.88	14.65 to 25.88	14.65 to 25.88	14.65 to 25.88	14.65 to 25.88	14.65 to 25.88
15	Goodness of Fit									
16	Degrees of Freedom									48
17	R square	0.8583	-0.0001727	0.4070	-2020	0.3498	0.7719	0.3007	-11.20	0.9526
18	Absolute Sum of Squares	34954	8473	33931	13719	15526	27868	67744	14415	216629
19	Sy.x									67.18
20	Constraints									
21	Vmax	Vmax is shared	Vmax is shared	Vmax is shared	Vmax is shared	Vmax is shared	Vmax is shared	Vmax is shared	Vmax is shared	
22	I	I = 0.0	I = 25.00	I = 100.0	I = 200.0	I = 250.0	I = 500.0	I = 1000	I = 2000	
23	Ki	Ki is shared	Ki is shared	Ki is shared	Ki is shared	Ki is shared	Ki is shared	Ki is shared	Ki is shared	
24	KM	KM is shared	KM is shared	KM is shared	KM is shared	KM is shared	KM is shared	KM is shared	KM is shared	
25	Number of points									
26	Analyzed	9	3	9	3	6	9	9	3	
27										

Bn-(*S*)-MeSpd

	Nonlin fit	A	B	C	D	E	F	G	H	I
		0	25	100	200	250	500	1000	2000	Global (shared)
		Y	Y	Y	Y	Y	Y	Y	Y	Y
1	Noncompetitive inhibition									
2	Best-fit values									
3	Vmax	1502	1502	1502	1502	1502	1502	1502	1502	1502
4	I	= 0.0	= 25.00	= 100.0	= 200.0	= 250.0	= 500.0	= 1000	= 2000	
5	Ki	155.0	155.0	155.0	155.0	155.0	155.0	155.0	155.0	155.0
6	KM	32.94	32.94	32.94	32.94	32.94	32.94	32.94	32.94	32.94
7	Std. Error									
8	Vmax	89.27	89.27	89.27	89.27	89.27	89.27	89.27	89.27	89.27
9	Ki	12.61	12.61	12.61	12.61	12.61	12.61	12.61	12.61	12.61
10	KM	5.471	5.471	5.471	5.471	5.471	5.471	5.471	5.471	5.471
11	95% Confidence Intervals									
12	Vmax	1322 to 1681	1322 to 1681	1322 to 1681	1322 to 1681	1322 to 1681	1322 to 1681	1322 to 1681	1322 to 1681	1322 to 1681
13	Ki	129.6 to 180.3	129.6 to 180.3	129.6 to 180.3	129.6 to 180.3	129.6 to 180.3	129.6 to 180.3	129.6 to 180.3	129.6 to 180.3	129.6 to 180.3
14	KM	21.93 to 43.95	21.93 to 43.95	21.93 to 43.95	21.93 to 43.95	21.93 to 43.95	21.93 to 43.95	21.93 to 43.95	21.93 to 43.95	21.93 to 43.95
15	Goodness of Fit									
16	Degrees of Freedom									48
17	R square	0.8261	-0.8254	0.7670	-24.79	0.5074	0.5576	-4.464	-34.94	0.9594
18	Absolute Sum of Squares	42894	4079	54084	11245	17851	45885	63940	15729	255707
19	Sy.x									72.99
20	Constraints									
21	Vmax	Vmax is shared	Vmax is shared	Vmax is shared	Vmax is shared	Vmax is shared	Vmax is shared	Vmax is shared	Vmax is shared	
22	I	I = 0.0	I = 25.00	I = 100.0	I = 200.0	I = 250.0	I = 500.0	I = 1000	I = 2000	
23	Ki	Ki is shared	Ki is shared	Ki is shared	Ki is shared	Ki is shared	Ki is shared	Ki is shared	Ki is shared	
24	KM	KM is shared	KM is shared	KM is shared	KM is shared	KM is shared	KM is shared	KM is shared	KM is shared	
25	Number of points									
26	Analyzed	9	3	9	3	6	9	9	3	

Bz-(*R*)-MeSpd

Nonlin fit	A	B	C	D	E	F	G
	0	100	250	500	1000	2000	Global (shared)
	Y	Y	Y	Y	Y	Y	Y
1 Noncompetitive inhibition							
2 Best-fit values							
3 Vmax	1141	1141	1141	1141	1141	1141	1141
4 I	= 0.0	= 100.0	= 250.0	= 500.0	= 1000	= 2000	
5 Ki	589.0	589.0	589.0	589.0	589.0	589.0	589.0
6 KM	13.41	13.41	13.41	13.41	13.41	13.41	13.41
7 Std. Error							
8 Vmax	57.86	57.86	57.86	57.86	57.86	57.86	57.86
9 Ki	56.77	56.77	56.77	56.77	56.77	56.77	56.77
10 KM	2.831	2.831	2.831	2.831	2.831	2.831	2.831
11 95% Confidence Intervals							
12 Vmax	1024 to 1258	1024 to 1258	1024 to 1258	1024 to 1258	1024 to 1258	1024 to 1258	1024 to 1258
13 Ki	474.4 to 703.6	474.4 to 703.6	474.4 to 703.6	474.4 to 703.6	474.4 to 703.6	474.4 to 703.6	474.4 to 703.6
14 KM	7.695 to 19.13	7.695 to 19.13	7.695 to 19.13	7.695 to 19.13	7.695 to 19.13	7.695 to 19.13	7.695 to 19.13
15 Goodness of Fit							
16 Degrees of Freedom							42
17 R square	0.8183	-0.04134	-0.09510	0.2597	0.4697	-5.174	0.9070
18 Absolute Sum of Squares	44813	71881	28650	54106	35553	29777	264780
19 Sy.x							79.40
20 Constraints							
21 Vmax	Vmax is shared	Vmax is shared	Vmax is shared	Vmax is shared	Vmax is shared	Vmax is shared	
22 I	I = 0.0	I = 100.0	I = 250.0	I = 500.0	I = 1000	I = 2000	
23 Ki	Ki is shared	Ki is shared	Ki is shared	Ki is shared	Ki is shared	Ki is shared	
24 KM	KM is shared	KM is shared	KM is shared	KM is shared	KM is shared	KM is shared	
25 Number of points							
26 Analyzed	9	9	6	9	9	3	
27							

Bz-(*S*)-MeSpd

Nonlin fit	A	B	C	D	E	F	G
	0	100	250	500	1000	2000	Global (shared)
	Y	Y	Y	Y	Y	Y	Y
1 Noncompetitive inhibition							
2 Best-fit values							
3 Vmax	1154	1154	1154	1154	1154	1154	1154
4 I	= 0.0	= 100.0	= 250.0	= 500.0	= 1000	= 2000	
5 Ki	846.0	846.0	846.0	846.0	846.0	846.0	846.0
6 KM	11.79	11.79	11.79	11.79	11.79	11.79	11.79
7 Std. Error							
8 Vmax	53.07	53.07	53.07	53.07	53.07	53.07	53.07
9 Ki	81.95	81.95	81.95	81.95	81.95	81.95	81.95
10 KM	2.422	2.422	2.422	2.422	2.422	2.422	2.422
11 95% Confidence Intervals							
12 Vmax	1047 to 1261	1047 to 1261	1047 to 1261	1047 to 1261	1047 to 1261	1047 to 1261	1047 to 1261
13 Ki	680.6 to 1011	680.6 to 1011	680.6 to 1011	680.6 to 1011	680.6 to 1011	680.6 to 1011	680.6 to 1011
14 KM	6.904 to 16.68	6.904 to 16.68	6.904 to 16.68	6.904 to 16.68	6.904 to 16.68	6.904 to 16.68	6.904 to 16.68
15 Goodness of Fit							
16 Degrees of Freedom							42
17 R square	0.7674	0.2974	0.2307	-0.8618	0.2090	-8.157	0.8930
18 Absolute Sum of Squares	57371	58628	30219	42190	59332	19757	267496
19 Sy.x							79.81
20 Constraints							
21 Vmax	Vmax is shared	Vmax is shared	Vmax is shared	Vmax is shared	Vmax is shared	Vmax is shared	
22 I	I = 0.0	I = 100.0	I = 250.0	I = 500.0	I = 1000	I = 2000	
23 Ki	Ki is shared	Ki is shared	Ki is shared	Ki is shared	Ki is shared	Ki is shared	
24 KM	KM is shared	KM is shared	KM is shared	KM is shared	KM is shared	KM is shared	
25 Number of points							
26 Analyzed	9	9	6	9	9	3	
27							

P4-(*R*)-MeSpd

Nonlin fit	A	B	C	D	E	F	G
	0	25	100	250	500	1000	Global (shared)
	Y	Y	Y	Y	Y	Y	Y
1 Noncompetitive inhibition							
2 Best-fit values							
3 Vmax	1178	1178	1178	1178	1178	1178	1178
4 I	= 0.0	= 25.00	= 100.0	= 250.0	= 500.0	= 1000	
5 Ki	1277	1277	1277	1277	1277	1277	1277
6 KM	16.45	16.45	16.45	16.45	16.45	16.45	16.45
7 Std. Error							
8 Vmax	36.78	36.78	36.78	36.78	36.78	36.78	36.78
9 Ki	110.7	110.7	110.7	110.7	110.7	110.7	110.7
10 KM	2.009	2.009	2.009	2.009	2.009	2.009	2.009
11 95% Confidence Intervals							
12 Vmax	1104 to 1252	1104 to 1252	1104 to 1252	1104 to 1252	1104 to 1252	1104 to 1252	1104 to 1252
13 Ki	1054 to 1500	1054 to 1500	1054 to 1500	1054 to 1500	1054 to 1500	1054 to 1500	1054 to 1500
14 KM	12.40 to 20.51	12.40 to 20.51	12.40 to 20.51	12.40 to 20.51	12.40 to 20.51	12.40 to 20.51	12.40 to 20.51
15 Goodness of Fit							
16 Degrees of Freedom							42
17 R square	0.8510	-2.724	0.9022	0.9217	0.8771	-1.985	0.9071
18 Absolute Sum of Squares	36753	1510	14388	2362	13046	76633	144692
19 Sy.x							58.69
20 Constraints							
21 Vmax	Vmax is shared	Vmax is shared	Vmax is shared	Vmax is shared	Vmax is shared	Vmax is shared	
22 I	I = 0.0	I = 25.00	I = 100.0	I = 250.0	I = 500.0	I = 1000	
23 Ki	Ki is shared	Ki is shared	Ki is shared	Ki is shared	Ki is shared	Ki is shared	
24 KM	KM is shared	KM is shared	KM is shared	KM is shared	KM is shared	KM is shared	
25 Number of points							
26 Analyzed	9	3	9	6	9	9	
27							

P4-(*S*)-MeSpd

Nonlin fit	A	B	C	D	E	F	G
	0	25	100	250	500	1000	Global (shared)
	Y	Y	Y	Y	Y	Y	Y
1 Noncompetitive inhibition							
2 Best-fit values							
3 Vmax	1215	1215	1215	1215	1215	1215	1215
4 I	= 0.0	= 25.00	= 100.0	= 250.0	= 500.0	= 1000	
5 Ki	1016	1016	1016	1016	1016	1016	1016
6 KM	15.21	15.21	15.21	15.21	15.21	15.21	15.21
7 Std. Error							
8 Vmax	36.77	36.77	36.77	36.77	36.77	36.77	36.77
9 Ki	79.12	79.12	79.12	79.12	79.12	79.12	79.12
10 KM	1.900	1.900	1.900	1.900	1.900	1.900	1.900
11 95% Confidence Intervals							
12 Vmax	1140 to 1289	1140 to 1289	1140 to 1289	1140 to 1289	1140 to 1289	1140 to 1289	1140 to 1289
13 Ki	856.4 to 1176	856.4 to 1176	856.4 to 1176	856.4 to 1176	856.4 to 1176	856.4 to 1176	856.4 to 1176
14 KM	11.38 to 19.05	11.38 to 19.05	11.38 to 19.05	11.38 to 19.05	11.38 to 19.05	11.38 to 19.05	11.38 to 19.05
15 Goodness of Fit							
16 Degrees of Freedom							42
17 R square	0.8394	-0.4236	0.4561	0.5112	0.8237	0.5858	0.9299
18 Absolute Sum of Squares	39617	8747	36964	14122	21386	26109	146945
19 Sy.x							59.15
20 Constraints							
21 Vmax	Vmax is shared	Vmax is shared	Vmax is shared	Vmax is shared	Vmax is shared	Vmax is shared	
22 I	I = 0.0	I = 25.00	I = 100.0	I = 250.0	I = 500.0	I = 1000	
23 Ki	Ki is shared	Ki is shared	Ki is shared	Ki is shared	Ki is shared	Ki is shared	
24 KM	KM is shared	KM is shared	KM is shared	KM is shared	KM is shared	KM is shared	
25 Number of points							
26 Analyzed	9	3	9	6	9	9	
27							
28							

Michaelis-Menten Graphs and Data of **Supplementary Material 1** Tables 1 and 2

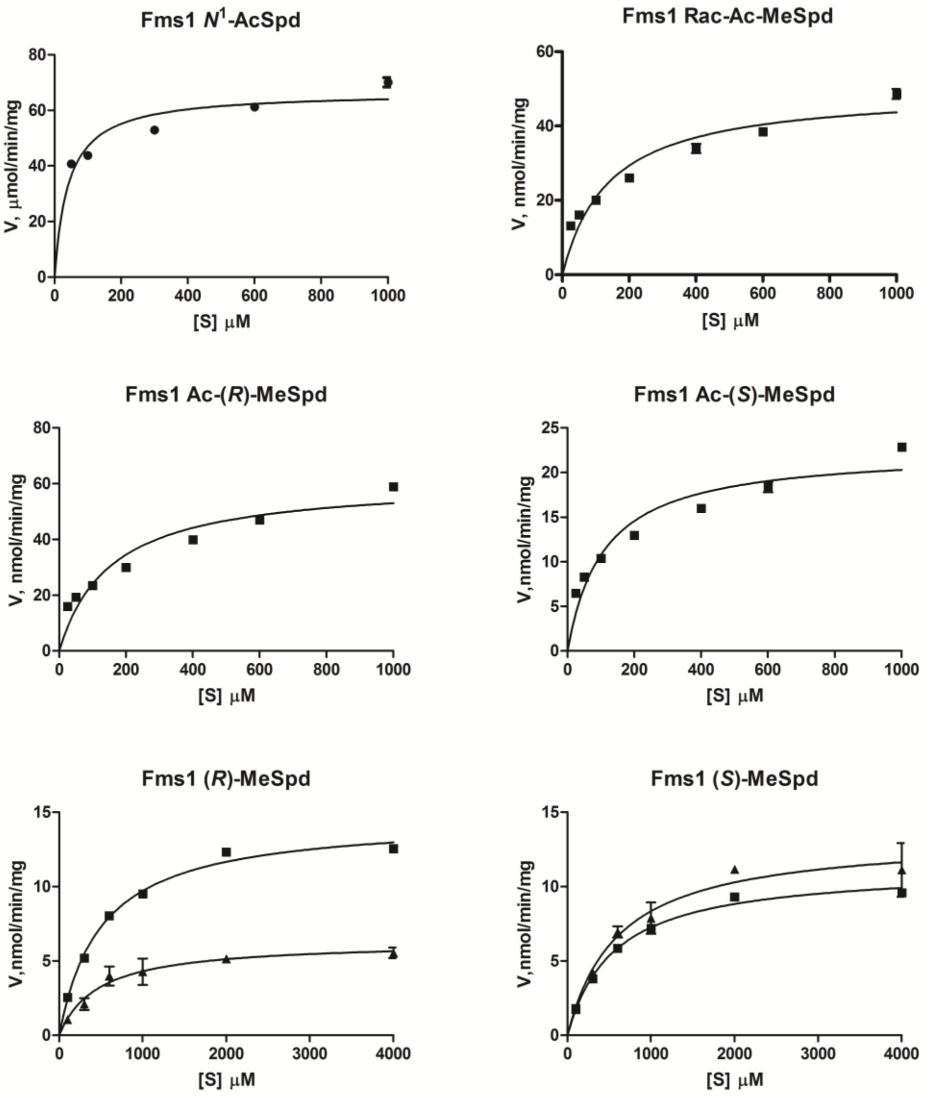

GraphPad Prism 5.03	*N*[1]AcSpd	Rac-AcMeSpd	Ac-(*R*)-MeSpd	Ac-(*S*)-MeSpd	(*R*)-MeSpd	(*R*)-MeSpd	(*S*)-MeSpd	(*S*)-MeSpd
	µmol/min/mg	nmol/min/mg	nmol/min/mg	nmol/min/mg	nmol/min/mg	nmol/min/mg	nmol/min/mg	nmol/min/mg
Michaelis-Menten					E1	E2	E1	E2
Best-fit values								
V_{max}	66.65	49.78	61.24	22.51	14.59	6.315	11.28	13.40
K_m	41.78	139.3	154.8	109.2	502.2	466.6	555.1	606.0
Std. Error								
V_{max}	2.294	2.568	3.584	1.054	0.2778	0.3535	0.2146	0.7203
K_m	7.564	23.43	28.59	17.97	31.11	87.16	33.19	99.42
95% Confidence Intervals								
V_{max}	61.69 to 71.60	44.41 to 55.15	53.74 to 68.74	20.31 to 24.72	14.00 to 15.18	5.565 to 7.064	10.83 to 11.74	11.87 to 14.92
K_m	25.44 to 58.12	90.22 to 188.3	94.97 to 214.6	71.63 to 146.8	436.2 to 568.1	281.8 to 651.4	484.7 to 625.4	395.3 to 816.8
Goodness of Fit								
Degrees of Freedom	13	19	19	19	16	16	16	16
R square	0.8226	0.9099	0.8962	0.9027	0.9898	0.9156	0.9909	0.9396
Absolute Sum of Squares	316.5	271.1	464.4	59.74	2.402	4.183	1.292	13.25
Sy.x	4.934	3.778	4.944	1.773	0.3874	0.5113	0.2842	0.9099
Constraints								
K_m	Km > 0.0	Km > 0.0	Km > 0.0	Km > 0.0	Km > 0.0	Km > 0.0	Km > 0.0	Km > 0.0
Number of points								
Analyzed	15	21	21	21	18	18	18	18
